# Transition to fetal calf serum-free culture enhances kidney proximal tubule cell bioenergetics and allows pharmacological applications

**DOI:** 10.1007/s00441-026-04072-7

**Published:** 2026-05-23

**Authors:** Thomas K. van der Made, Pim Cleijpool, Polly Paul, Devon A. Barnes, Stefan Oswald, Bonnie C. Broeksma, Quentin Faucher, Silvia M. Mihăilă, Rosalinde Masereeuw

**Affiliations:** 1 Department of Pharmaceutical Sciences, Pharmacology, Utrecht University, Universiteitsweg 99, 3584 CG Utrecht, The Netherlands; 2 Institute of Pharmacology and Toxicology, Rostock University Medical Center, Rostock, 18057 Germany; 3 Danone Nutricia Research, Utrecht, The Netherlands

**Keywords:** Drug transporters, Pharmacology, Reduction of animal use

## Abstract

Proximal tubule in vitro systems are important tools in pre-clinical drug development, providing insights into drug disposition and safety. Cells are often cultured in medium containing fetal calf serum (FCS); however, FCS addition is not physiologically relevant for cells of human origin. Here, in vitro cultures of human kidney proximal tubule cells were transitioned to FCS-free medium. The impact of FCS-free medium on maturation and maintenance for multiple passages of commonly used conditionally immortalized proximal tubule epithelial cells overexpressing the pharmacologically-relevant organic anion transporter 1 (ciPTEC-OAT1) was evaluated. This transport protein is lost upon isolation and in vitro culture of tubule cells, and due to its clinical relevance, an OAT1 expressing cell line was chosen. Supplementation of FCS-free medium with human platelet lysates supported longer-term cell growth. OAT1-mediated fluorescein transport was sensitive to probenecid inhibition under both conditions, although intracellular fluorescein accumulation was reduced in FCS-free cultures. KEGG and GO enrichment analysis of differentially expressed genes revealed that inflammatory response and HIF-1 signaling were downregulated, whereas cell adhesion and ATP-binding cassette transporter genes were upregulated in FCS-free cultures. Interestingly, an increase in bioenergetic profile was observed for ciPTEC-OAT1 cultured in optimized FCS-free medium. Furthermore, in 3D bioengineered kidney tubule cultures, supplementation with an increased concentration of human platelet lysates lead to improved cell viability and fiber coverage. To conclude, we highlighted significant efforts and strategies in transitioning FCS-containing to FCS-free medium for ciPTEC-OAT1 cultures.

**Supplementary Information:**

The online version contains supplementary material available at 10.1007/s00441-026-04072-7.

## Introduction

In vitro kidney proximal tubule cultures are important tools to gain mechanistic insights into drug disposition and to facilitate toxicity screening during pre-clinical drug development (Stresser et al. 2023). The kidney is an important excretory organ involved in the clearance of many endo- and exogenous compounds (Morrissey, et al. 2013), making accurate and robust in vitro systems to predict renal clearance important for pharmacokinetic/pharmacodynamic applications (Fowler, et al. 2020; Phillips, et al. 2020). However, to maintain the growth and maturation, cells within these systems are often cultured in medium containing fetal calf serum (FCS), a complex mixture of growth factors, proteins, vitamins, trace elements, hormones, lipids and other components (van der Valk, et al. 2010). Supplementation of culture medium with FCS has several disadvantages. Firstly, the composition of FCS is undefined and subject to high batch-to-batch variability, impacting data reproducibility (van der Valk, et al. 2010). Secondly, ethical concerns with the harvest and use of FCS have been raised. Better defined and more human-relevant FCS-free medium is hypothesized to be beneficial in developing a universal medium that supports long-term maintenance of multiple human cell types in advanced in vitro models, such as multi-organ-on-chip systems (Oleaga, et al. 2016; Sasserath, et al. 2020). Hence, there is a clear need for a more defined FCS-free medium.

Prior studies have focused on transitioning proximal tubule cells to FCS-free medium (see Tab. S1). Considerations for successful transition to FCS-free cell cultures include medium composition, extracellular matrix coatings to support cell adherence and growth (Dankers, et al. 2011; Zhang, et al. 2009) and implementation of an adaptation phase for cells to these new conditions (van der Valk, et al. 2010). It is important to account for the complex cell- and in vitro system-specific environment that FCS supports, including cell attachment, growth and survival, protection against shear forces and toxic compounds developing over time in the medium (Meng and Day 2026). Development of a fully defined FCS-free medium likely requires extensive and high-throughput screening of various compounds in different concentrations that support cell growth and general viability. Additionally, other less defined substances, such as serum albumin, are important medium additions to support cell viability and functionality (Francis 2010). Alternatively, studies have also highlighted that sources of human origin, such as human platelet lysates (hPL) or human serum, can serve as direct replacements for FCS in various cell types (Burnouf, et al. 2016, Wanes, et al. 2021).

The present study focuses on the kidney proximal tubular cells, as they constitute the main site of drug handling and are highly sensitive to drug-induced nephrotoxicity. Various cell sources can be utilized for in vitro proximal tubule systems (Bajaj, et al. 2018), including a conditionally immortalized proximal tubule epithelial cell (ciPTEC) line. This cell line has been utilized to study renal toxicity and drug transport in 2D and 3D in vitro systems (Jansen, et al. 2016; Nieskens, et al. 2016; Vriend, et al. 2018), either as the parent cell line or with the organic anion transporter 1 (ciPTEC-OAT1) or 3 (ciPTEC-OAT3) transfected. Both transporters accept a wide range of endo- and exogenous substrates, including drugs and their metabolites (Russel, et al. 2002), but are rapidly lost upon culturing (Van der Hauwaert, et al. 2014). While the ciPTEC-OAT1/3 lines have proven to be valuable tools in drug screens, transition to FCS-free conditions has yet to be achieved. In this study, the focus was on the OAT1 transporter as an important, clinically relevant proximal tubule drug transporter that is often lost upon in vitro culture.

FCS-free culture conditions for ciPTEC-OAT1 cells were established after thorough optimization and functional evaluation in a step-wise process: First, the impact of FCS-free medium added to ciPTEC-OAT1 during the maturation phase cultured in 2D was assessed. Next, the composition of FCS-free medium was optimized with supplementation with various types of hPL to support longer-term ciPTEC-OAT1 culture (> 5 passages). The performance of ciPTEC-OAT1 in 2D-optimized FCS-free medium, compared to the currently used FCS-containing medium, was studied by evaluating differences in general viability and cellular morphology, changes in genome wide transcriptomics, differences in the expression and abundance of relevant drug transporters and mitochondrial activity. Finally, experimental conditions for ciPTEC-OAT1 cultured in FCS-free 3D conditions were optimized by evaluating ciPTEC-OAT1 cell-material interaction, growth and metabolic activity.

## Materials and methods

Materials were purchased from Merck Life Science (Amsterdam, The Netherlands) unless stated otherwise. Concentrations listed in round brackets are final concentrations. A more detailed description of the methods is available in the Supplemental Methods.

### Composition of FCS-containing and FCS-free medium formulations

Tab. S1 gives an overview of studies that have reported FCS-free medium formulations for culture of proximal tubule cell sources, which served as background information for this study. DMEM-F12 (ThermoFisher Scientific, Breda, The Netherlands) was supplemented with 10% FCS (Merck Life Science, Amsterdam, The Netherlands, VWR, Amsterdam, The Netherlands), insulin–transferrin–sodium selenite (ITS, 5 µg/mL, 5 µg/mL, 5 ng/mL), epidermal growth factor (10 ng/mL), hydrocortisone (36.1 ng/mL) and tri-iodothyronine (40 pg/mL), further referred to as FCS-containing medium. Advanced DMEM-F12 (ThermoFisher Scientific, Breda, The Netherlands, catalog #12634010) was supplemented with GlutaMAX™ (1 ×, ThermoFisher Scientific, Breda, The Netherlands), HEPES (10 mM, ThermoFisher Scientific, Breda, The Netherlands), epidermal growth factor (50 ng/mL), hydrocortisone (36.1 ng/mL) and tri-iodothyronine (40 pg/mL), further referred to as FCS-free medium. hPLs (ELAREM™ Prime or ELAREM™ Perform, PL Biosciences, Aachen, Germany) were added together with heparin (2 U/mL), as per manufacturer’s recommendation, to FCS-free medium, further referred to FCS-free medium + X% hPL. Addition of individual supplements to FCS-free medium, e.g. prostaglandin E1 (25 ng/mL), B-27™ supplement (1 ×, Thermofisher Scientific, Breda, The Netherlands), poloxamer 188 non-ionic surfactant (0.1%, Thermofisher Scientific, Breda, The Netherlands) was tested.

### ciPTEC-OAT1 culture in 2D experiments to evaluate the impact of FCS-free medium on maturation

Human ciPTEC-OAT1, originally isolated from a healthy human urine sample (Wilmer, et al. 2010) and transduced to express OAT1 (Nieskens, et al. 2016) were provided by Cell4Pharma (Oss, The Netherlands; www.cell4pharma.com) with the ethical approval and quality assurance in place. For evaluation of various FCS-free medium supplements in part I, ciPTEC-OAT1 were maintained in the proliferative phase at 33 °C in FCS-containing medium under static conditions before seeding (between passage number 50–60). CiPTEC-OAT1 were seeded (63,000 cells/cm^2^) in a 24-well plate using FCS-containing medium. After 24 h at 33 °C, medium was replaced with FCS-containing or FCS-free medium and cells were matured at 37 °C for 7 days (medium was refreshed once every other day). For evaluation of various coatings in part I, ciPTEC-OAT1 (reaching 90% confluency in a flask with FCS-containing medium) were adapted to FCS-free medium for 4 days prior to cell seeding. Details on coating procedures of 24-well plates can be found in supplemental methods. CiPTEC-OAT1 were cultured for at least one passage in optimized FCS-free medium.

### Evaluation of general cell viability by PrestoBlue assay in 2D experiments

On day 6 of maturation at 37 °C, ciPTEC-OAT1 monolayers were evaluated microscopically. Medium was aspirated and PrestoBlue (ThermoFisher Scientific, Amsterdam, The Netherlands) reagent mixed with cell culture medium (1:10 ratio) was added (500 µL/well). After 1 h in the dark at 37 °C, 100 µL of medium was transferred to a 96-well plate and fluorescence (520 nm excitation, 580–640 nm emission) was measured using GloMax® Discover Microplate Reader (Promega, Leiden, The Netherlands, GM3000). Remaining solution was aspirated; cells were washed once with HBSS and the fresh respective medium was added. Triton X-100 (1%) was used as a control for limited cell viability.

### Evaluation of OAT1 functionality by intracellular fluorescein uptake in 2D experiments

On day 7 of maturation at 37 °C, ciPTEC-OAT1 monolayers were evaluated microscopically. Cells were washed with warm HBSS (+ 10 mM HEPES, pH 7.4, further referred to as HBSS in this section). To evaluate OAT1 functionality, cells were incubated with fluorescein (1 µM, OAT1 substrate) ± probenecid (100 µM, prototypical OAT1 inhibitor) in the dark at 37 °C for 10 min. Then, incubation medium was aspirated swiftly and cells were washed 1 × with cold HBSS. Cells were lysed by adding 0.1 M NaOH (250 µL/well) and placed on a rocker (IKA Vibrax VXR, VWR, Amsterdam, The Netherlands) for 10 min, in dark. Thereafter, fluorescence (GloMax® Discover Microplate Reader, 475 nm excitation, 500–550 nm emission) was measured. A fluorescein calibration curve (range 0.0025–10 µM in 0.1M NaOH) was included to convert absolute fluorescence units to a concentration (µM). Cell lysate samples were stored at − 20 °C. Protein amount was determined using the BCA protein quantification kit (ThermoFisher Scientific, Breda, The Netherlands) according to manufacturer’s protocol. The amount of protein determined was used to normalize the PrestoBlue and fluorescein uptake data.

### Evaluation of cell growth using various medium formulations

Briefly, cells were seeded at a density of 13,000 cells/cm^2^ in 24-well plates. CiPTEC-OAT1 were proliferated in FCS-free medium conditions with various concentrations of ELAREM™ Prime (1, 5, 10%), ELAREM™ Perform (1%) human platelet lysates, or FCS-containing medium. Surface coverage and cell morphology were evaluated microscopically and cells were counted (automated LUNA-II™ cell counter, Westburg, Leusden, The Netherlands) on day 7 when cultured over multiple cell passages. FCS-free medium + 1% ELAREM™ Perform was further selected as condition for evaluating cell growth in T25 and T75 culture flasks. Either 0.33 × 10^6^ (T25) or 1 × 10^6^ (T75) cells were seeded in the flask on day 0. Cells were detached (using accutase) and counted on days 4, 7, and 10 for cell growth determination. To assess long-term culture, cells were detached and counted on day 7 and subsequently 1 × 10^6^ cells were seeded in a new T75 flask.

### Evaluation of ciPTEC-OAT1 transcriptomics in 2D FCS-free and FCS-containing cultures

CiPTEC-OAT1 were maintained for 1 or 5 passages on FCS-free medium + 1% ELAREM™ Perform or in FCS-containing medium as control. Cells were seeded in a 6-well plate and cultured for 1 day at 33 °C and at 37 °C for 7 days. Cells were lysed in 1000 µL RLT (Qiagen, Venlo, The Netherlands) buffer with 10 µL β-ME and stored at − 80 °C until further analysis. RNA isolation, quality control of RNA in samples, library prep (Truseq RNA stranded polyA), using Illumina NextSeq2000 (P3: 2 × 50 bp) was performed by USEQ facility (see supplemental methods). Data was analysed using iDEP software (software version 1.13, Ge, Son & Yao, iDEP, BMC Bioinformatics 19:1–24, 2018 (Ge, et al. 2018)) by inputting three replicates per condition individually (conditions: FCS_P1; FCS_P5; FCSfree_P1; FCSfree_P5). To filter out genes with low counts, the minimum counts per million was set at 0.5 in at least 2 samples. Differential expression was calculated with a false discovery rate (FDR) of 0.05 using DESeq2. Enrichment *P*-values are calculated based on one-sided hypergeometric test, which is then adjusted for multiple testing using the Benjamini–Hochberg procedure and converted to FDR. Fold Enrichment is defined as the percentage of genes in the list belonging to a pathway, divided by the corresponding percentage in the background. The background genes are filtered genes from the original gene list. The filtered genes are those that passed a low filter in RNA-seq. A total of 347 gene sets was obtained from KEGG (Kyoto Encyclopedia of Genes and Genomes). Database version/date is Release 104.0. Furthermore, genesets are derived from GOBP, GOCC and GOMF. After the analysis is done, pathways are first filtered based on a FDR cutoff (0.05). Then the significant pathways are sorted by FDR. Only the top 5 pathways are shown. Mean read counts for FCS and FCS-free conditions (pooled passages) are accessible online (https://esbl.nhlbi.nih.gov/Databases/ciPTEC-OAT1/).

### Determination of transporter protein abundances in 2D ciPTEC-OAT1 cultures

A T75 flask was coated with 10 mL Collagen Type IV (25 µg/mL) in HBSS at RT for 1 h, covered with aluminum foil. CiPTEC-OAT1 cells were seeded at a density of 63,000 cells/cm^2^, subsequently cultured at 33 °C for 1 day, followed by 7 days at 37 °C. Cells were harvested, washed 3 × with HBSS and subsequently lysed in 1 mL ice-cold lysis buffer (0.2% SDS, 5 mM EDTA, supplemented with 5 µL/mL Protease Inhibitor Cocktail Set III). Samples were homogenized (VWR, Precellys 24) followed by a centrifugation step (15,294 × g for 10 min at 4 °C). Samples were incubated for 30 min at 4 °C while shaking at 100 rpm and stored at − 80 °C until further analysis. Samples were further prepared (Drozdzik, et al. 2019 and supplemental methods) and protein quantification was measured by mass spectrometry-based targeted proteomics (Oswald, et al. 2019).

### Evaluation of cellular bioenergetics by Seahorse XF cell mito stress test

The oxygen consumption rate (OCR) and extracellular acidification rate (ECAR) were determined using Seahorse XF mito stress test (Agilent Technologies, Amstelveen, The Netherlands). The mito stress test was done according to manufacturer’s instructions and details can be found in supplemental methods. Briefly, XF96-well microplates (ref # 103793–100) were coated with Collagen Type IV solution (25 µg/mL) at RT in the dark for 1 h. Various ciPTEC-OAT1 seeding densities in FCS-containing (6 or 12 × 10^3^ cells/well) or FCS-free + 1% ELAREM™ Perform (6, 12 or 18 × 10^3^ cells/well) were investigated. ciPTEC-OAT1 were incubated for 1 day at 33 ºC and at 37 °C for 7 days. ciPTEC-OAT1 monolayers were evaluated prior to the assay using brightfield microscopy (Fig. S1). Oligomycin (1.5 µM) and Rotenone/Antimycin (0.5 µM) concentrations were selected according to Agilent Seahorse XF Cell Mito Stress guidelines. In contrast, FCCP concentration (2.0 µM) was based on a previously performed titration experiment for ciPTEC14.4 (Faria et al. 2023). The sensor cartridges were loaded into the Seahorse Analyzer and calibration was performed. Subsequently, after successful calibration, the utility plate was removed, the cell loaded plate was inserted and the program (Wave software version 2.6.8.3) started. After the run, the cells were lysed in 50 µL of RIPA (ThermoFisher Scientific, Amsterdam, The Netherlands) buffer for subsequent protein analysis by BCA.

### Evaluation of bioengineered kidney tubules by culturing ciPTEC-OAT1 on hollow fiber membranes

Hollow fiber membranes (HFMs) from Baxter (Hechingen, Germany) with an inner diameter of 320 µm and wall thickness of 50 µm were cut at a length of 10 mm. HFMs were sterilized, coated and seeded according to the previously described protocol by Jansen et al. (2016) with some changes (see supplemental methods). CiPTEC-OAT1 cells were expanded in their respective medium, e.g. FCS-containing or FCS-free medium (+ 1% ELAREM Perform hPL), for at least 1 passage prior to seeding on the HFMs (Fig.S2). Medium was refreshed every other day. When flasks were > 90% confluent, the cells were washed 1 × with HBSS and detached using Accutase (Sigma Aldrich, A6964) for ~ 5 min at 33 ºC. Cell suspension was added to Eppendorf tubes containing 3–4 coated HFMs and incubated at 33 ºC for 4 h. Eppendorf tubes were rotated every hour to ensure cell adhesion on all surfaces of the HFM. After seeding, HFMs were transferred to a 24-well plate (1 HFM per well) containing 500 μl medium. CiPTEC-OAT1-HFM were evaluated microscopically at least 4 h after seeding, after proliferation phase and prior to experiments. Using a sterile tweezer, ciPTEC-OAT1-HFMs were transferred to another 24-well plate (1 HFM/well) with 500 µL/well PrestoBlue reagent mixed with respective cell culture medium (1:10 ratio). Of note, for all FCS-free test conditions, during the 1 h incubation with PrestoBlue reagent/cell culture medium, FCS-free medium + 1% ELAREM™ Perform was used. The same procedure was followed as during the 2D experiments. After 7 days of maturation at 37 °C, ciPTEC-OAT1-HFMs were fixed and stained with phalloidin and DAPI (see details in supplemental methods). Fluorescent images and Z-stacks were acquired by confocal microscopy (Leica TCS SP8 X imaging system) and reconstructed in Fiji.

### Statistical analysis

**Fig. 1:**
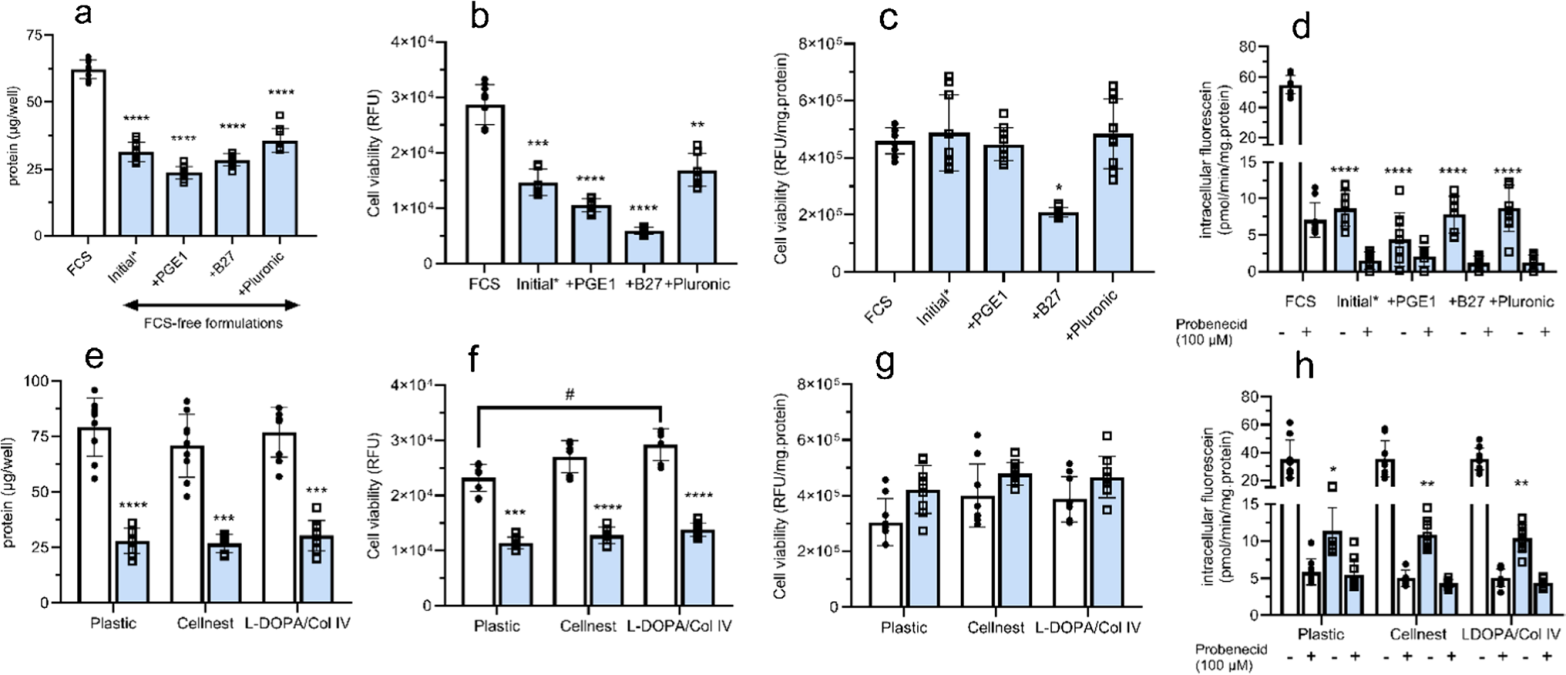
Impact of medium additions (**a**–**d**) or extracellular matrix coating (**e**–**h**) on ciPTEC-OAT1 protein content, cell viability and OAT1-mediated drug transport. ciPTEC-OAT1 were cultured in FCS-containing (white bar,) or FCS-free medium (**blue** bar,). **a–d** Cells were seeded using FCS-containing medium and after 24 h at 33 °C, medium was replaced with FCS-containing or FCS-free medium and cells were matured at 37 °C for 7 days. **e–h** ciPTEC-OAT1 were adapted to FCS-free medium for 4 days prior to cell seeding, after seeding ciPTEC-OAT1 were cultured in FCS-containing or FCS-free medium. **a, e** Protein content measured in cell lysates from 24-well plates after fluorescein assay. **b, f** Cell viability determined by relative fluorescence units (RFU). **c, g** Cell viability after normalization to protein content. **d, h** OAT1-mediated intracellular accumulation of OAT1 substrate fluorescein (1 µM) ± OAT1 inhibitor probenecid (100 µM) after 10 min incubation. Depicted datapoints ± SD are *N* = 3 with 3 technical replicates per experiment. * = *p* < 0.05, ** = *p* < 0.01, *** = *p* < 0.001, **** = *p* < 0.0001, # = *p* < 0.05 for FCS-containing with respect to difference between coating condition. No statistics were performed for the probenecid conditions in **d, h**. “Initial*” in panel **a–d** refers to FCS-free medium as defined in the method section, which was also used as FCS-free medium in panel **e–h**. One datapoint was excluded in the FCS-free + B27 group without inhibitor in panel d due to being a clear outlier (40.1 compared to 7.8 pmol/min/mg.protein for the average of the other 8 datapoints)

Graphs and statistical analyses were performed using GraphPad Prism (GraphPad Software version 9.0.0, San Diego, California USA, www.graphpad.com). Data are depicted as mean ± SD, with all biological and technical replicates highlighted. Background fluorescence was subtracted for PrestoBlue and fluorescein experiments. Agilent Seahorse Analytics was used for data acquisition of Seahorse data by inputting the asyr file. GraphPad Prism was used for further Seahorse data analysis and presentation. IDEP (software version 1.13, Ge, Son & Yao, iDEP, BMC Bioinformatics 19:1–24, 2018) was used to analyse RNA sequencing reads, 3 replicates per condition have been inputted individually in iDEP 1.13 (see Supplemental Methods). Statistics were done with GraphPad Prism, using the biological replicates only. For Fig. 1a–d, an ordinary one-way ANOVA and Dunnett’s multiple comparisons test was performed to compare FCS-free medium with the FCS-containing control. F values represent the ratio of variance between groups to variance within groups obtained from ANOVA. For Fig. 1e–h, Fig. 6a, b and Fig. 7a, b, a two-way ANOVA was performed with Bonferroni’s multiple comparisons test to compare FCS-free medium with the FCS-containing control. In Fig. 6a, the mean of the conditions were compared with the control (e.g. FCS-containing 1 × 10^6^ cells/mL) mean. Two-tailed unpaired *t*-test was performed in Figs. 4c and 5c. Normal distribution and equal SD were assumed. No statistics were performed for the probenecid conditions in Figs. 1d, h and 4c.

## Results

### Culturing ciPTEC-OAT1 in FCS-free medium resulted in reduced cell density and OAT1 functionality, without compromising cell viability

The impact of replacing FCS-containing medium with FCS-free medium using various supplements (Fig. 1a–d) or extracellular matrix coatings (Fig. 1e–h) during the maturation phase was assessed. Significant differences in cell density, based on protein content, were evident between FCS-containing and FCS-free cultures (Fig. 1a, F (4, 10) = 58.37,* p* < 0.0001), which aligned with microscopic observations (Fig. S3). Normalization of ciPTEC-OAT1 cell viability data (Fig. 1b) to protein content (Fig. 1c) revealed comparable values between FCS-containing and FCS-free medium cultures, except for cultures supplemented with B27 in FCS-free medium, which showed reduced cell viability. Both FCS-containing and FCS-free cultures showed functional OAT1-mediated fluorescein uptake, which was sensitive to inhibition by probenecid; however, intracellular accumulation of fluorescein was significantly decreased in FCS-free cultures (Fig. 1d). Differences in protein content were also evident between FCS-containing and FCS-free cultures for all coating conditions (Fig. 1e, F (1, 12) = 105.0, *p* < 0.0001), although these coatings did not significantly affect this nor cell viability overall (Fig. 1e–g). Intracellular accumulation of fluorescein was decreased by 3.1–3.3-fold in FCS-free cultures for all extracellular matrix coatings compared to FCS-containing cultures (Fig. 1h, F (1, 12) = 39.66, *p* < 0.0001). These findings suggested that ciPTEC-OAT1 can be matured in FCS-free culture medium on 2D well plates. However, cells stopped expanding after one passage in FCS-free medium (Fig. S4, DMEM-F12 and ADMEM-F12 0% conditions) and OAT1 activity was reduced.

### Addition of human platelet lysates to FCS-free medium supports longer-term expansion and passaging of ciPTEC-OAT1 cultures

**Fig. 2:**
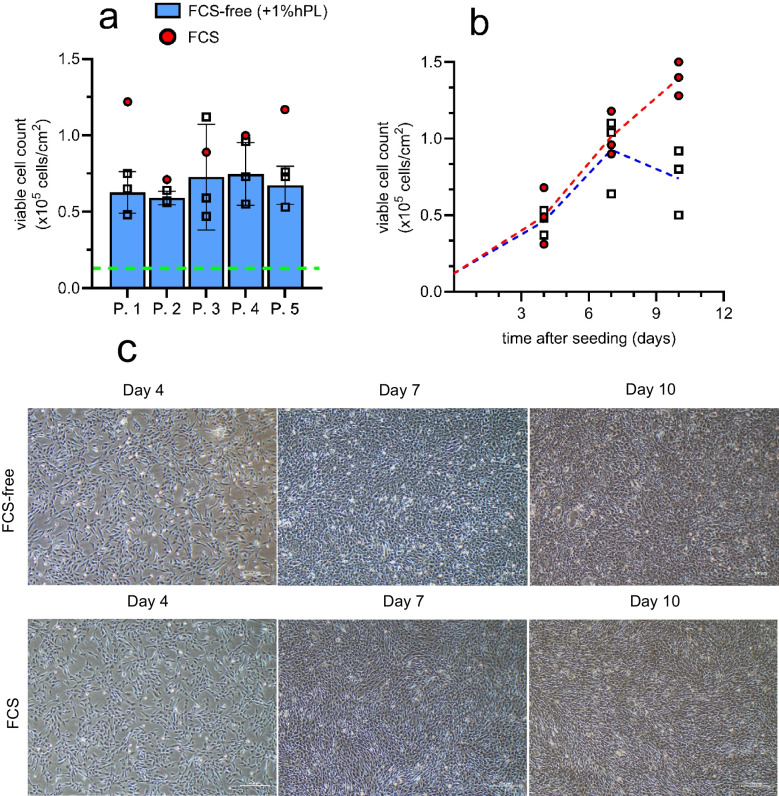
Evaluation of long-term ciPTEC-OAT1 cell expansion in FCS-containing or FCS-free medium (+ 1% ELAREM Perform™).** a** Number of viable cells per cm^2^ for multiple passages (P.#) at day 7 after transition to FCS-free cell culture (blue bar,) or the FCS control (  ). An initial seeding of 1.0 × 10^6^ cells per T75 (0.13 × 10^5^ viable cells/cm^2^) was used (green dashed line). **b** Viable cells per cm^2^ at designated time for FCS-free (  ) and FCS (  ) cell cultures. An initial seeding of 3.0 × 10^5^ cells per T25 (0.12 × 10^5^ viable cells/cm^2^) was used. The red and blue dashed lines connect the mean values of FCS and FCS-free, respectively, between days. **c** Representative microscopic image of ciPTEC-OAT1 morphology and cell confluency at day 4, 7 and 10 after seeding. Scale bar = 200 µm. Depicted datapoints are from *N* = 3 using 1 replicate, except for the FCS condition in 2 a (*N* = 1)

The impact of replacing FCS-containing medium with various FCS-free medium formulations on cell growth was assessed in a pilot study (Fig. S4). Based on these results, FCS-free medium supplemented with 1% ELAREM Perform™ (hPL) was selected as the optimized FCS-free medium for subsequent 2D experiments. Optimized FCS-free medium enabled consistent ciPTEC-OAT1 cultures for at least five passages on this medium, though cell counts on day 10 were lower compared to those from FCS-containing conditions (Fig. 2).

### ciPTEC-OAT1 transcriptomics analysis reveals substantial gene expression changes after culture in optimized FCS-free medium

**Fig. 3:**
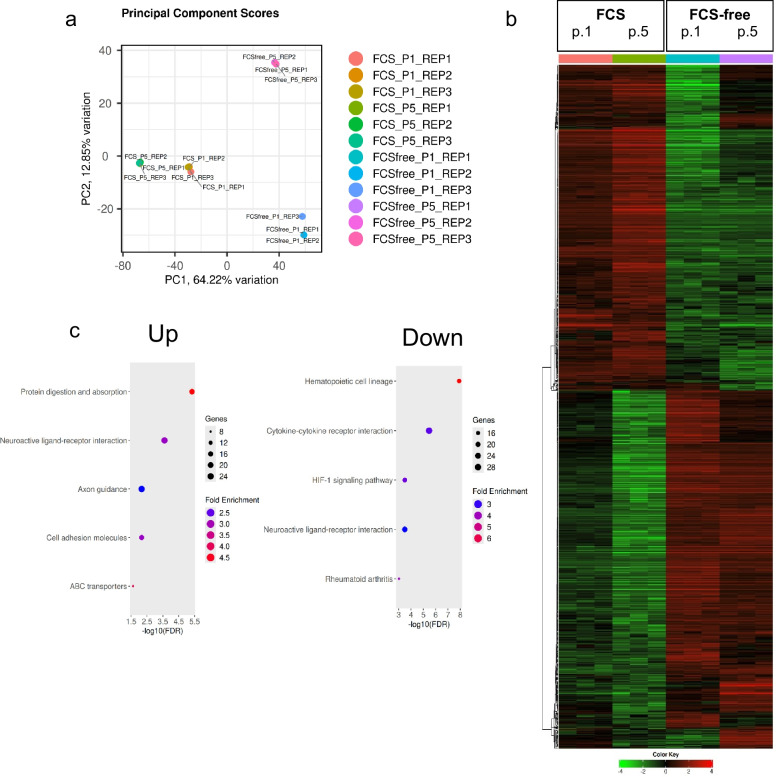
Genome wide transcriptome analysis and transcriptomics differences between FCS-containing and optimized FCS-free cultures. **a** Principal component analysis (PCA) for clustering of ciPTEC-OAT1 samples expanded in optimized FCS-free medium for 1 or 5 passages or their respective FCS-control. **b** Hierarchical clustering for different conditions of top 1000 most variable genes. **c** Up- and downregulated pathways in FCS-free compared to FCS-containing cultures for Kyoto Encyclopedia of Genes and Genomes (KEGG), for this analysis data on passages have been combined. *N* = 1 performed in triplicates is shown for each condition (conditions: FCS_P1; FCS_P5, FCSfree_P1; FCSfree_P5). Three replicates per condition have been inputted individually in iDEP 1.13 (see Supplemental Methods)

Transcriptomic analysis was performed to identify genome-wide expression changes and changes in related pathways after transition to optimized FCS-free medium for 1 or 5 passages. The PCA plot revealed distinct clustering between FCS-containing and FCS-free ciPTEC-OAT1 cultures on PC1, with most of the variation being captured by PC1 and PC2 (Fig. 3a). Overall, gene expression trends were similar between the FCS-free culture passages and between the FCS-containing culture passages, as highlighted by hierarchical clustering (Fig. 3b). For subsequent analyses (Fig. 3c), samples from passages 1 and 5 after transition to optimized FCS-free medium were pooled, and the same was done for the FCS-containing control, to focus on pathways and genes consistently up- or downregulated between medium conditions; 1183 differentially expressed genes were upregulated and 1071 differentially expressed genes were downregulated in FCS-free medium. Top downregulated genes in FCS-free cultures based on -log10 adjusted p-value (Tab. S2) included HIF-1α-regulated genes (*BNIP3* (Bhargava and Schnellmann 2017) and *PPFIA4 (*Wang, et al. 2005*)*), genes related to cellular response to hypoxia (*PDK1 (*Schley, et al. 2012*)*), extracellular matrix related genes (*COL6A3 (*Zeng, et al. 2019*)* and *FN1 (*Waalkes, et al. 2010*)*), rate-limiting enzymes for glycolysis (*HK2 (*Lan, et al. 2016*)*), and the genes *PLOD2* and *DUXAP9*. Upregulated genes included *SPP1* and *JAG1*. Upregulated pathways in FCS-free cultures identified in GO:BP all related to cell and cell–cell adhesion pathways (Fig.S5). Cell adhesion molecules were also identified in KEGG (Fig. 3c). The inflammatory response and responses to external stimuli pathways were downregulated in FCS-free cultures, as identified in GO:BP (Fig.S5). Finally, ABC transporters were identified as upregulated in FCS-free cultures, whereas HIF1 signaling pathway was downregulated (Fig. 3c). Gene expression results in this study have not been confirmed in individual gene expression assays.

### Decreased OAT1 expression and function with increased expression of efflux drug transporters in cells cultured in optimized FCS-free medium

**Fig. 4:**
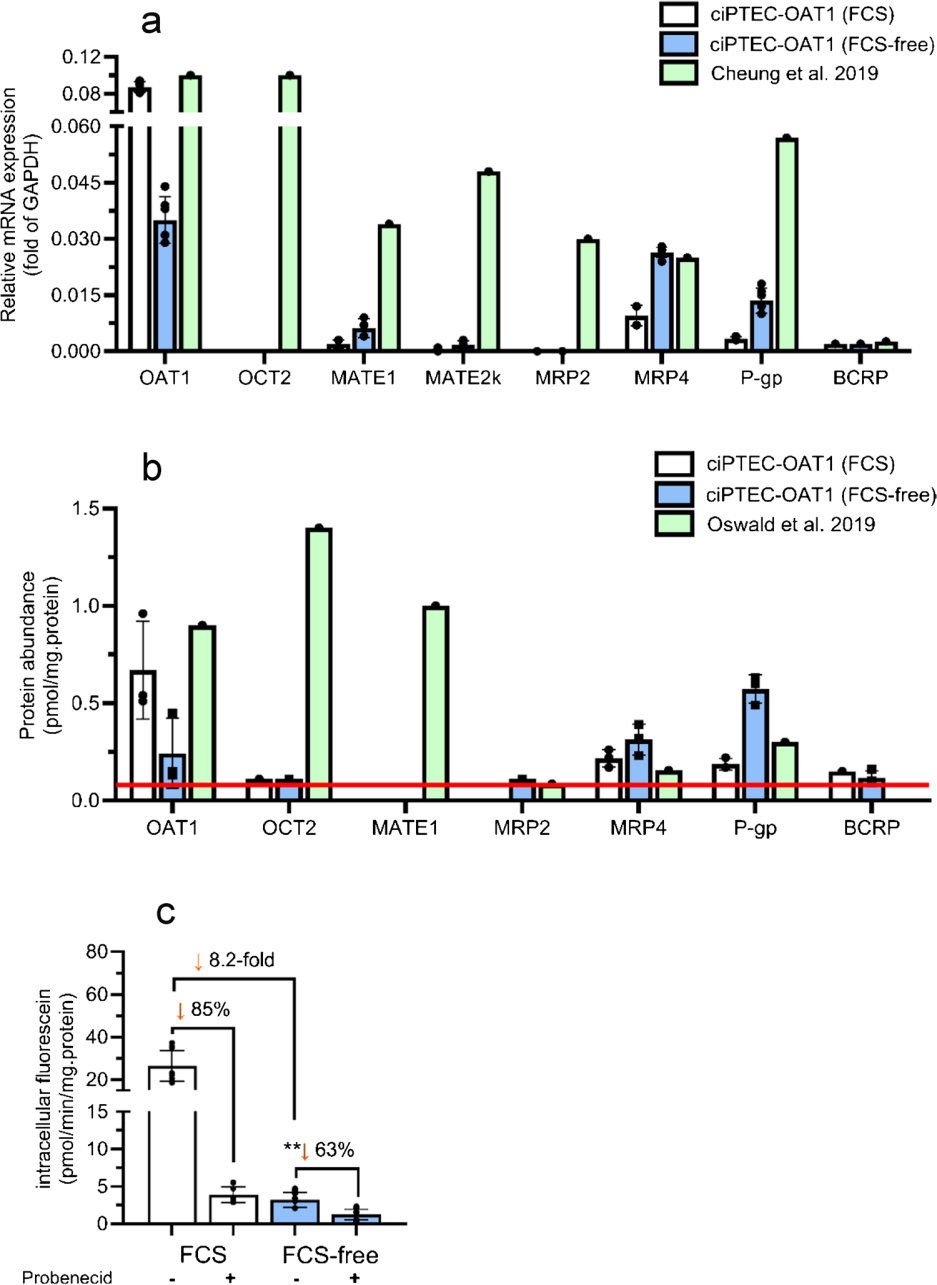
Gene expression (**a**) and protein abundance (**b**) of clinically relevant renal drug transporters and OAT1 transporter functionality (**c**) for ciPTEC-OAT1 cultured in FCS-containing or optimized FCS-free medium.** a** Relative mRNA expression (fold of GAPDH) of proximal tubule drug transporters in ciPTEC-OAT1 cultured in FCS-containing (□) or FCS-free (  ). For this analysis, data from RNA-seq for passage 1 and 5 were pooled (*N* = 2 in triplicates). Note that GAPDH expression was twofold decreased for FCS-free cultures compared to FCS-containing cultures (146000 vs 74000 read counts). Relative mRNA expression of drug transporters (median fold of GAPDH) in renal cortical tissues in adults, reported by Cheung et al. (2019), are highlighted in  . **b** Transporter protein abundance in ciPTEC-OAT1 (cultured in FCS-containing or optimized FCS-free medium). Literature using human renal kidney cortices (data reported by (Oswald, et al., 2019)) is also highlighted. MATE1 was < LLOQ in ciPTEC-OAT1, 2 out of 3 samples were < LLOQ for OCT2 for both medium conditions. Transporter protein abundance for 3 out of 3 and 1 out of 3 were < LLOQ for MRP2 for FCS and optimized FCS-free medium, respectively. LLOQ = 0.05 nmol/L (  ). Data from Oswald et al. was extracted using plotdigitizer. Data is shown for *N* = 3 with 1 replicate per independent experiment. **c** OAT1-mediated intracellular accumulation of OAT1 substrate fluorescein (1 µM) ± OAT1 inhibitor probenecid (100 µM) after 10 min incubation. Depicted datapoints are from *N* = 3 in triplicates ** = *p* < 0.01. No statistics were performed for the probenecid conditions. ciPTEC-OAT1 cells were expanded in their respective medium, e.g. FCS-containing or FCS-free medium (+ 1% ELAREM Perform hPL) for at least 1 passage prior to seeding

The International Transporter Consortium (Galetin, et al. 2024) reported clinically relevant proximal tubule transporters that should be tested in drug development. The expression, based on RNA-seq (Fig. 4a), and protein abundance (Fig. 4b) of these transporters were evaluated. For both medium conditions, the rank order in relative mRNA expression in ciPTEC-OAT1 (Fig. 4a) was: OAT1 > MRP4 > P-gp > MATE1 > BCRP > MATE2k > MRP2, whereas OCT2 and OAT3 were not detected. The rank order for protein abundance of transporters in ciPTEC-OAT1 cultured in FCS-containing medium (Fig. 4b) was OAT1 > MRP4 > P-gp > BCRP > OCT2/MRP2/MATE1 (< LLOQ for several/all samples). For optimized FCS-free medium (Fig. 4b), this rank order changed to P-gp > MRP4 > OAT1 > BCRP > MRP2/OCT2/MATE1 (< LLOQ for several/all samples). OAT1 expression and protein abundance for cells cultured in FCS-containing medium were closer to values reported for renal cortical tissues in the literature (Fig. 4) (Cheung, et al. 2019, Oswald, et al. 2019). Interestingly, relative OAT1 mRNA expression and protein abundance decreased by 2.5 and 2.7 fold for ciPTEC-OAT1 cultured in optimized FCS-free medium compared to FCS-containing medium, respectively. Notably, GAPDH expression was twofold decreased for FCS-free cultures compared to FCS-containing cultures (146000 vs 74000 read counts, respectively). Furthermore, OAT1 activity, as determined by the intracellular accumulation of OAT1 substrate fluorescein, appeared 8.2-fold decreased in FCS-free cultures compared to FCS-containing cultures (Fig. 4c). The expression of genes reported to be potentially involved in OAT downregulation is highlighted in Fig.S6a (Caetano-Pinto and Stahl 2023). Genes consistently showing a ≥ twofold difference in FCS-free compared to FCS-containing were: *GLUT3* (downregulated), *Ahr* (upregulated) and *HNF1ß* (upregulated). Additionally, *HDAC7* gene expression was 1.4-fold upregulated for cells in FCS-free medium (Fig. S6a). Conversely, several efflux transporters were increased in ciPTEC-OAT1 cultured in optimized FCS-free medium, such as MRP4 gene expression (2.8-fold) and protein abundance (1.5-fold), and P-gp gene expression (4.1-fold) and protein abundance (3.1-fold).

### Optimized FCS-free ciPTEC-OAT1 cultures show improved bioenergetic phenotype and comparable cell viability

**Fig. 5:**
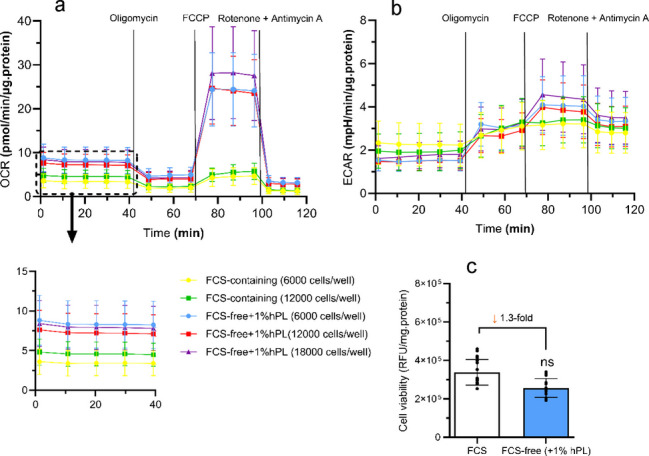
Improved bioenergetic phenotype and comparable cell viability for ciPTEC-OAT1 cultured in optimized FCS-free medium. Mitochondrial respiration indicators **a** oxygen consumption rate (OCR) and **b** extracellular acidification rate (ECAR) were measured before and after injections of Oligomycin, FCCP and Rotenone + Antimycin A and normalized to protein. Data is shown for initial ciPTEC-OAT1 cell seeding densities and different media conditions: FCS-containing: 6 × 10^3^ cells/well (  ), 12 × 10^3^ cells/well (  ) and optimized FCS-free: 6 × 10^3^ cells/well (  ), 12 × 10^3^ cells/well (  ) and 18 × 10^3^ cells/well (  ). Mean ± SD of *N* = 3 with 12 technical replicates per experiment; 15 values were excluded in total across all conditions based on issues with Oligomycin, FCCP, Rotenone + Antimycin A injection and/or ECAR measurements. **c** Absolute fluorescence units normalized to mg protein in the well measured in PrestoBlue assay. Depicted datapoints are from *N* = 3 performed in sixplicates. ciPTEC-OAT1 cells were expanded in their respective medium, e.g. FCS-containing or FCS-free medium (+ 1% ELAREM Perform hPL) for at least 1 passage prior to seeding

Mitochondrial respiration, assessed using the Seahorse Mito Stress Test, was evaluated for ciPTEC-OAT1 cultured in FCS-containing and optimized FCS-free medium (Fig. 5). For most studied parameters, differences between various initial cell seeding densities were minor in both medium conditions (Fig. 5,Fig. S7 and Tab. S3). ciPTEC-OAT1 cultured in optimized FCS-free medium had a 5 and 13.5 -fold higher maximal respiration and spare respiratory capacity compared to cells cultured in FCS-containing medium, respectively, when seeding was performed at 12,000 cells/ well (Tab. S3). In addition, basal respiration, ATP-production coupled respiration and non-mitochondrial oxygen consumption were significantly increased for ciPTEC-OAT1 cultured in optimized FCS-free medium compared to FCS-containing medium, albeit to a lesser extent (Tab. S3,Fig. S7). An increase in ECAR (glycolytic parameter) was evident for all conditions and initial cell seeding densities after blocking ATP synthase with oligomycin (Fig. 5b). Rate-based measurements of respiration and extracellular pH can broadly characterize an energetic phenotype (Fig. S8). The difference between baseline phenotype and stressed phenotype (*e.g.* first measurement after FCCP injection) is highest for the ciPTEC-OAT1 cultured in optimized FCS-free medium. ciPTEC-OAT1 cell viability was slightly decreased for optimized FCS-free medium cultures after normalization to protein content (Fig. 5c). KEGG pathways (Kanehisa 2019; Kanehisa, et al. 2025; Kanehisa and Goto 2000) for glycolysis, oxidative phosphorylation and mitophagy demonstrate differences in gene expression between optimized FCS-free and FCS-containing cultures (Fig. S9). A non-exhaustive list of genes implicated in key events for kidney injury markers, oxidative stress, mitochondrial dysfunction and inflammation are shown in Fig. S6c, as highlighted in a recent review by Barnes et al. (Barnes, et al. 2024).

### Higher percentage of human platelet lysates improves cell viability and coverage of bioengineered kidney tubules in FCS-free cultures

**Fig. 6:**
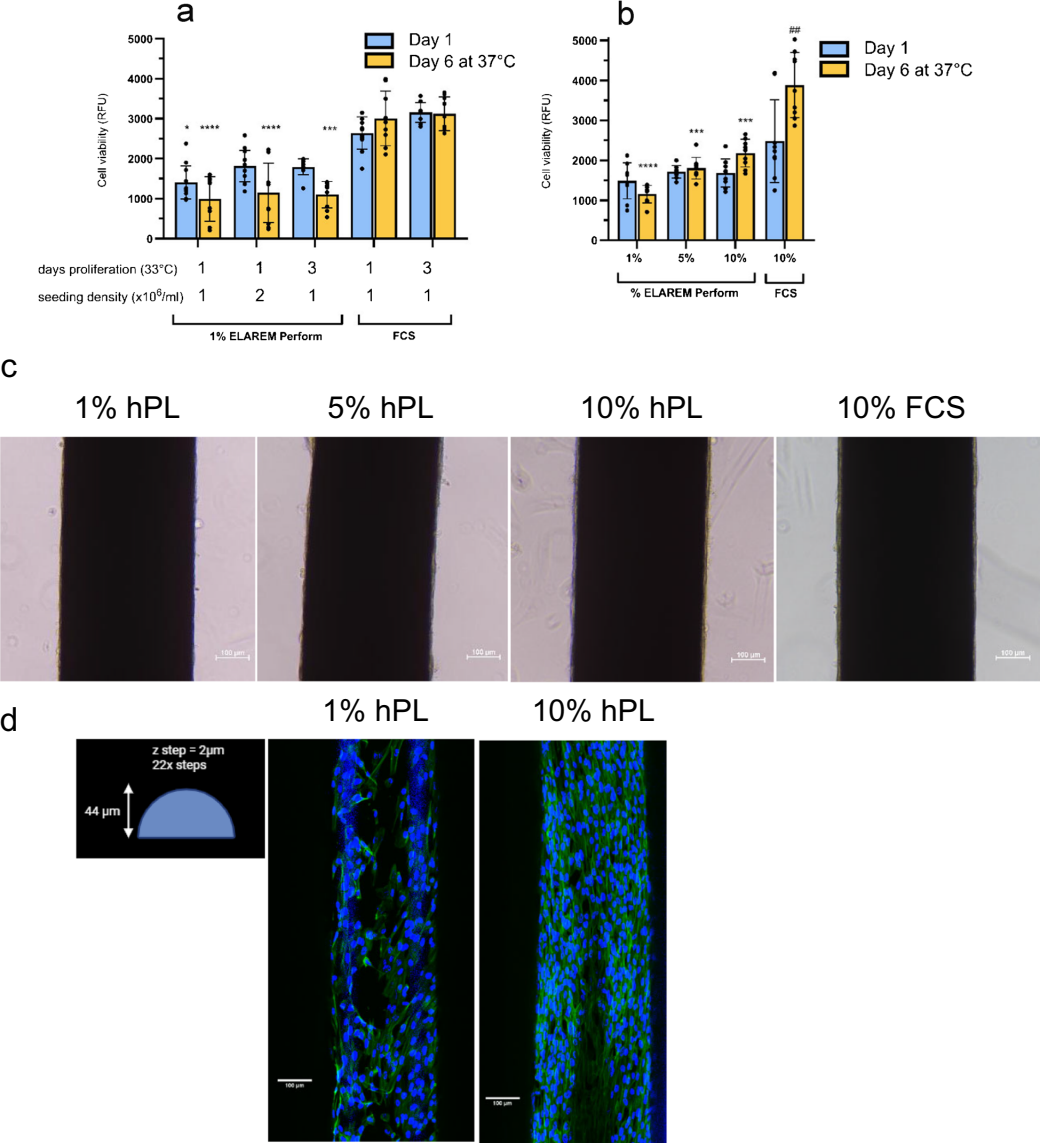
Effect of various experimental conditions on FCS-free culture on bioengineered kidney proximal tubule formation.** a** Impact of the number of proliferative days (1 vs 3) and cell seeding density (1 vs 2 × 10^6^ cells/mL) on cell viability measurements after proliferation (blue bar) and maturation (orange bar) phase (*N* = 3). **b** Impact of ELAREM™ Perform titration in FCS-free medium on cell viability measurements (*N* = 3). A cell seeding density of 2 × 10^6^ cells/mL for FCS-free cultures and 1 day at 33 °C were used, **c** cell attachment by brightfield and **d** cell coverage after maturation by immunofluorescent staining. **c** Representative microscopic image of ciPTEC-OAT1-HFM per condition after maturation. **d** Representative confocal z-stack image of ciPTEC-OAT1-HFM for 1% and 10% hPL condition after maturation showing DAPI and phalloidin staining of ciPTEC-OAT1-HFM. Scale bar = 100 µm. * = *p* < 0.05, *** = *p* < 0.001 **** = *p* < 0.0001, between FCS and FCS-free. ## = *p* < 0.01 between day 1 and day 6 at 37 °C for FCS-containing

To support cell coverage and viability of ciPTEC-OAT1 on HFMs under static conditions, further enhancement of the optimized FCS-free medium for 2D experiments was required. Initially, the impact of increased cell seeding density or increased proliferation period on ciPTEC-OAT1 cell metabolism and HFM coverage was assessed using the 2D-optimized FCS-free medium (see Fig. S2 for workflow). After proliferation (day 1), metabolic activity was slightly increased (1.3-fold) for HFMs seeded at a higher seeding density (Fig. 6a); however, this diminished over 6 days of maturation. Thereafter, no differences in metabolic activity between the cell densities were observed, and values did not reach levels similar to the metabolic activity for cells in FCS-containing medium (day 6, Fig. 6a, F (1, 11) = 51.00, *p* < 0.0001). Similar trends were observed with an increased proliferation period (Fig. 6a). Based on the marginal benefit of higher seeding density, 2 × 10^6^ cells/ml were used for all subsequent HFM FCS-free experiments (see Fig.S2 for workflow). On the other hand, a dose–response increase in metabolic activity and general cell coverage over 6 days of maturation was evident for higher % hPL conditions (Fig. 6b), whereas metabolic activity was similar at day 1. Bioengineered kidney tubules cultured in 10% hPL medium had 2.1-fold increased metabolic activity than those cultured in medium containing 1% hPL (Fig. 6b), supported by morphology (Fig. 6c) and immunostaining (Fig. 6d). These brightfield images highlight that the bioengineered kidney tubules cultured in a higher % hPL exhibit a consistent layer of cells along the side of the HFM, comparable to the FCS-containing condition. Therefore, 10% hPL FCS-free medium was used for HFM coating and dynamic culture condition experiments.

### Dynamic culture conditions slightly improve bioengineered kidney tubules in FCS-free cultures

**Fig. 7:**
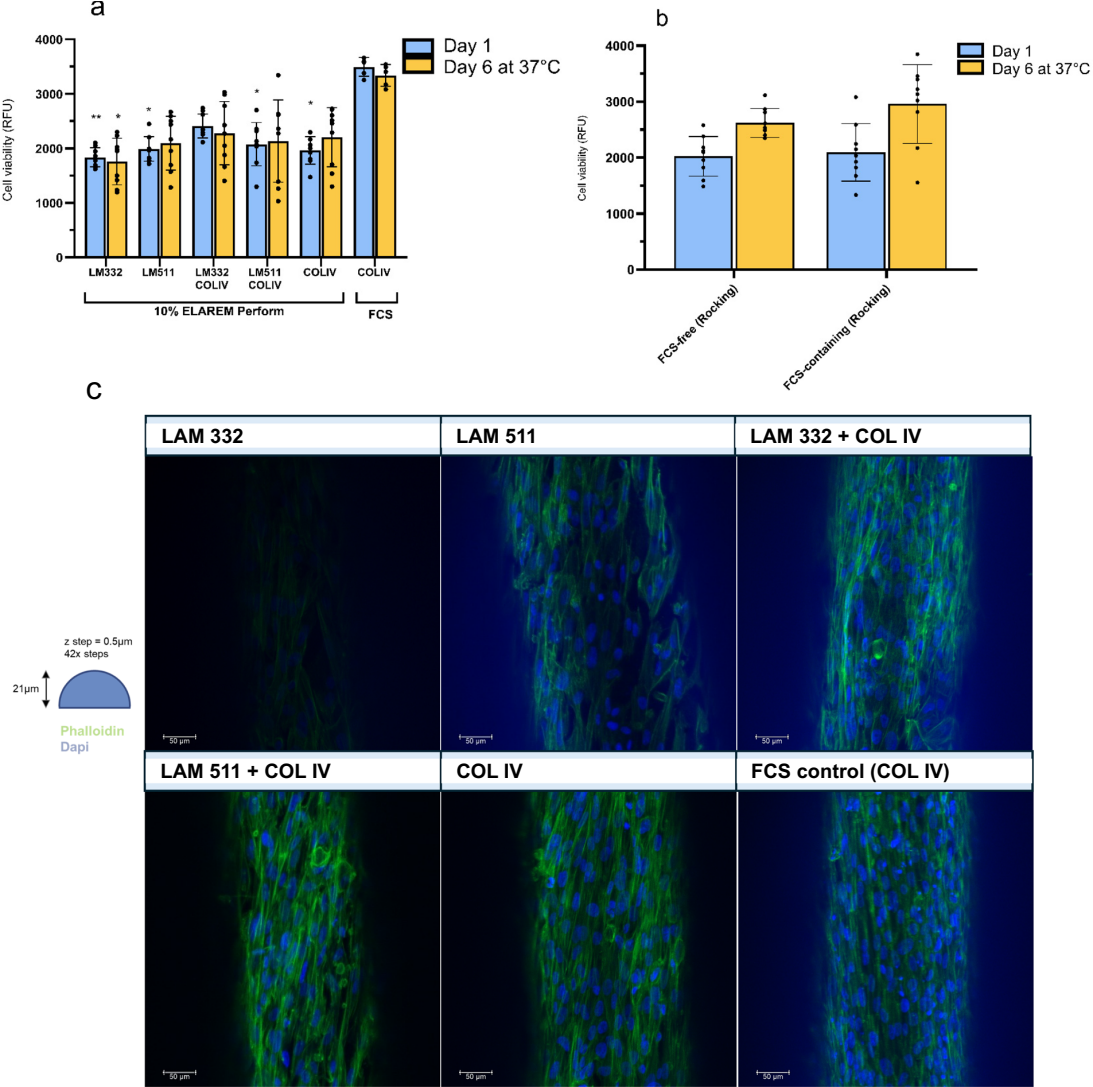
Effect of extracellular matrix and dynamic culture conditions on ciPTEC-OAT1-HFM.** a** Impact of extracellular matrix coating on cell viability measurements after proliferation (blue bar) and maturation (orange bar) phase. *N* = 3 and *N* = 2 for FCS-free and FCS-containing cultures, respectively. **b** Cell viability measurements after proliferation (blue bar) and maturation (orange bar) phase are highlighted for dynamic cultures (*N* = 3). Addition of 10% hPL, a cell seeding density of 2 × 10^6^ cells/mL for FCS-free cultures and 1 day at 33 °C were used for coating and rocking experiments. **c** Representative confocal z-stack image of ciPTEC-OAT1-HFM per condition after maturation showing DAPI and phalloidin staining of ciPTEC-OAT1-HFM. Scale bar = 50 µm. * = *p* < 0.05, ** = *p* < 0.01

The effects of various extracellular matrix coatings for the bioengineered tubules were assessed. After proliferation, there was increased metabolic activity observed for bioengineered kidney tubules coated with a combination of laminin-332 + collagen IV compared to the collagen IV only condition (Fig. 7a, 2341 vs 1999 RFU, respectively). However, this effect was not maintained after 6 days of maturation (Fig. 7a). Good cell coverage was maintained in 10% hPL FCS-free conditions as evaluated by confocal microscopy (Fig. 7c). Statistically significant differences in metabolic activity between the FCS control group and FCS-free groups were evident for day 1 (One-way ANOVA: *F* (5, 11) = 18.56, *p* < 0.0001), but not for 6 days of maturation (One-way ANOVA: *F* (5, 11) = 1.890). When kidney proximal tubules were proliferated for 1 day in static culture, then exposed to dynamic culture conditions using a 3D rocker during the 6-day maturation period, a 19% (2619 vs 2191 RFU) increase in metabolic activity was observed compared to those matured in static culture (Figs. 6b, 7a for static (*N* = 6) and Fig. 7b for dynamic culture (*N* = 3)). Nevertheless, the metabolic activity of the kidney proximal tubules under dynamic culture conditions was 85% of that from the tubules cultured in FCS-containing medium (Fig. 7b).

## Discussion

The ciPTEC cell line has previously been utilized to study renal toxicity and drug transport in 2D and 3D in vitro systems (Jansen, et al. 2016; Nieskens, et al. 2016; Vriend, et al. 2018); however, these cells are routinely cultured in FCS-containing medium. Therefore, cell cultures in FCS-containing medium have been chosen for comparison in this study. This study described a step-wise approach to transitioning ciPTEC-OAT1 to FCS-free medium that supports cell growth, viability and functionality. This is a step towards a serum-free screening system. Additionally, another potential application for serum-free ciPTEC-OAT1 cultures could be as a cell source in a bioengineered artificial kidney (Jansen, et al. 2016).

Our findings revealed that 2D ciPTEC-OAT1 FCS-free cultures show decreased cell density and decreased OAT1 functionality. General cell metabolic activity was found to be comparable between FCS and FCS-free medium conditions after normalization to protein content, a step included to account for potential differences in cell density. Notably, normalization of data based on protein content assumes that the amount of total protein per cell is comparable, which should be confirmed in future studies. Furthermore, the presence of FCS, hPL, or various concentrations of bovine serum albumin in the medium could influence the measured relative fluorescence units (*e.g.* by quenching of resorufin produced by cells). Therefore, direct comparison of relative fluorescence units between medium conditions to indicate cellular metabolic activity should be done with caution. Activity of the renal drug transporter OAT1 was confirmed in both media conditions by probenecid-sensitive intracellular accumulation of fluorescein; however, activity was greatly reduced in FCS-free cultures.

The addition of hPL to FCS-free medium supported longer-term (> 5 cell passages) cell growth, whereas cell growth in FCS-free medium without the addition of hPL was limited (< 2 cell passages). Human platelet lysates store a variety of potent substances that support cell expansion, such as EGF, PDGF, VEGF, FGF and HGF (Burnouf, et al. 2016; Rauch, et al. 2011). Furthermore, our study highlighted that use of ADMEM-F12 as basal medium improved cell expansion over 5 passages compared to DMEM-F12. One important difference between the two basal media is the addition of AlbuMAX™ II (*i.e.* lipid-rich bovine albumin, 400.0 mg/L) to ADMEM-F12. Albumin has multiple important functions, including binding and transporting physiologically relevant factors (such as lipids, metal ions, amino acids and signaling molecules), antioxidant properties, and maintenance of blood oncotic pressure and pH (Francis 2010). Rauch et al. (Rauch, et al. 2011) demonstrated comparable growth curves of the renal epithelial cell lines MDCK and LLC-PK1 using FCS or hPL supplementation over 4–7 days, whereas FCS-free medium without supplementation could not support cell growth, in line with the results in this study. Of note, successful development of a chemically defined culture medium, as has been done for example for MDCK (Taub, et al. 1979) and primary proximal tubule (Taub and Livingston 1981) cells, is desired as hPL preparations are also incompletely defined and subject to batch variation (Burnouf, et al. 2016). In addition, ethical and availability concerns also exist for hPL. Compared to FCS, however, it is markedly more ethical and physiologically relevant a supplement because it is human-derived, harvested with given consent and it can be produced from expired transfusable platelets (Burnouf, et al. 2016). Furthermore, chemically defined culture medium likely requires extensive and time-consuming testing, which was outside the scope of the current study. The impact of these supplement combinations on endpoints other than general cell viability and cell number has not been reported.

Transcriptomic analysis was performed to investigate potential mechanisms underlying the observed decrease in OAT1 activity and to explore differentially expressed gene pathways in optimized FCS-free cultures that could be meaningful for pharmacological and toxicological applications. Interestingly, HIF-1 signaling was downregulated in optimized FCS-free cultures. HIF-1 activation, induced by ROS and inflammation, promotes the expression of genes regulating glycolysis, cell survival and angiogenesis, as a response to hypoxic conditions (Taylor and Scholz 2022). HIF-mediated metabolic adaptations include diminished mitochondrial activity and fatty acid oxidation, whereas glycolysis is enhanced (Taylor and Scholz 2022). Indeed, genes related to glycolysis (glycolysis and glucose transporters) and mitophagy were downregulated, whereas genes related to fatty acid metabolism were upregulated in optimized FCS-free cultures (Fig. S6b). Together with the cellular bioenergetics measurements described below, a more oxidative phenotype is suggested for optimized FCS-free cultures. Interestingly, Du et al. highlighted that addition of 5% hPL induced a widespread transcriptome change for human fetal bone marrow mesenchymal stem cells, among which HIF-1 signaling pathway was downregulated in hPL cultures compared to FCS cultures (Du et al. 2021). They reported that HIF1A gene and protein expression were downregulated in hPL cultures, and it was hypothesized that this leads to a more oxidative phenotype (Du et al. 2021). More mechanistic studies into the HIF protein differences between cells cultured under both medium conditions would be useful, considering that HIF could influence injury or disease-mediated changes in metabolic processes in proximal tubule cells (Taylor and Scholz 2022).

Genes related to inflammatory response and cytokine-cytokine receptor interactions were found to be downregulated in optimized FCS-free medium. Exposure to a high concentration of inflammatory factors, which is assumed to be higher in 10% FCS compared to 1% hPL, will alter the stress response of statically cultured cells (Liu, et al. 2023). Liu et al*.* found that IL-8, but not TNFα or IL-1ß, was variably upregulated through the pERK pathway by supplementation of medium with FCS using the HCT-8 and HT-29 cell lines. Merrick et al. found that HEPHL1, COL6A3 and SERPINB2, which were Differentially Expressed Genes (DEGs) in our RNAseq dataset, were among the highest 20 DEGs at certain timepoints in mouse renal proximal tubule epithelial cells after repeated exposure with nephrotoxicant cisplatin (Merrick, et al. 2023). While we previously reported that ciPTEC-OAT1 acquire a senescent phenotype upon prolonged culturing in FCS-containing medium, differences in some identified genes were evident in our RNA-seq dataset between medium conditions (Fig. S6c and Yang et al. 2022). Future studies should compare the ciPTEC-OAT1 transcriptome with the transcriptome of proximal tubular cells in vivo. In this way, it could be determined if optimized FCS-free ciPTEC-OAT1 cultures carry a transcriptome phenotype more similar to that of proximal tubular cells in vivo (Shao, et al. 2024).

The observed reduced OAT1 expression and activity in optimized FCS-free cultures were supported by a reduced protein abundance, whereas expression and protein abundance of several drug efflux transporters were increased. This study highlights that ciPTEC-OAT1 cells can be matured in a FCS-free environment without losing all OAT1 functionality, although a substantial decrease in OAT1 expression, abundance and functionality was observed. A recent comprehensive review by Caetano-Pinto and Stahl (Caetano-Pinto and Stahl 2023) explored the complex regulatory mechanisms that govern the expression and activity of OAT1. For example, protein kinase C activation was found to inhibit human OAT1 activity through accelerated internalization of the transporter from the cell surface to intracellular compartments (Zhang, et al. 2008). In addition, HNF-4α, but not HNF-1α/ß, regulated human OAT1 gene expression (Ogasawara, et al. 2007). Furthermore, indoxyl sulfate has been found to activate Aryl hydrocarbon receptor by stimulating EGFR, which increased OAT1 expression (Jansen, et al. 2019). Interestingly, Caetano Pinto et al. discussed that cetuximab downregulated OAT1 and BCRP, but upregulated MRP4 and P-gp, in ciPTEC-OAT1, predominantly through ERK-mediated signaling downstream of EGFR (Caetano-Pinto, et al. 2017). The downregulated gene expression of EGFR and NFKß in optimized FCS-free cultures observed in this study could explain, at least in part, the observed downregulation of OAT1 (Fig. S6a). Of note, the ciPTEC-OAT1 cells originally generated by Nieskens et al. (Nieskens, et al. 2016) were derived from transduction of primary PTECs with a lentivirus driving expression of OAT1. This vector drives OAT1 expression via a strong constitutive promoter (CMV) (Mihajlovic, et al. 2019). Thus, any conclusions drawn from the changes in media composition cannot be translated directly to primary proximal tubule cells.

Concentrations of TCA cycle metabolites, in particular the intracellular concentration and efflux of α-ketoglutarate, a co-substrate for OAT1, could influence transporter activity; therefore, potential differences in their concentrations between medium conditions should be investigated in future studies (Caetano-Pinto and Stahl 2023; Vriend, et al. 2019). The increased gene expression and protein abundance of several efflux transporters for optimized FCS-free cultures could be related to EGFR signaling, as mentioned previously. Follow-up studies are also required to study OAT1 transport for a wider range of clinically relevant OAT1 substrates, such as furosemide (Stopfer, et al. 2018), and determine kinetic parameters. The substantial decrease in OAT1 activity in optimized FCS-free cultures could be a disadvantage for applications focusing on OAT1-mediated drug transport and assessment of sensitivity to nephrotoxicants that rely on OAT1 function to enter the cell (Sakolish, et al. 2025). Restoring OAT1 function in optimized FCS-free cultures should therefore be a priority for follow-up studies, to ensure that the cells are fit for purpose. This is needed to study the effects of other compounds on transporter function and to evaluate nephrotoxicity of compounds that are OAT1 substrates.

Comparison of the rank order of uptake- and efflux transporter abundance between in vitro and in vivo models could be considered for evaluating physiological relevance. Determining a rank order between OAT1, MRP4, P-gp and BCRP, all of which were above the determined LLOQ, could aid in determining the translational value of kinetic data obtained for ciPTEC-OAT1. Two studies using primary proximal tubule cells highlighted that OAT1 was most abundantly expressed of these 4 drug transporters, followed by P-gp and MRP4, whereas BCRP was below LLOQ (Oswald, et al. 2019, Prasad, et al. 2016). While OAT1 was most abundantly expressed in FCS-containing medium, this was not the case for optimized FCS-free cultures (3rd in rank order).

Optimized FCS-free ciPTEC-OAT1 cultures showed a more bioenergetic phenotype. Renal proximal tubule cells are highly metabolically active and require a significant amount of energy to perform their functions (Bhargava and Schnellmann 2017). However, a shift in energy production, favoring glycolysis, is often seen after in vitro culture of proximal tubule cells (Darshi, et al. 2023; Hammoud, et al. 2025). This shift has been suggested as one of the contributors to the loss of OAT expression and function in proximal tubule cells cultured using traditional in vitro methods (Caetano-Pinto and Stahl 2023). Previous studies have highlighted an oxidative phenotype for ciPTEC-OAT1 (Faria, et al. 2023; Hoogstraten, et al. 2023; Vriend, et al. 2019). van der Stel et al. demonstrated that differentiated RPTEC/TERT1 cells showed a more oxidative phenotype, independently of the presence of glucose, whereas 2D HepG2 cells showed higher sensitivity to mitochondrion-toxic compounds after switching glucose to galactose (van der Stel, et al. 2020). The slight discrepancy in bioenergetic phenotype could be related to differences in % of FCS in the medium (*e.g.* 0.5% versus 10% in our study) or could be cell type, protocol or system-specific (van der Stel, et al. 2020).

Higher spare respiratory capacity was shown for ciPTEC-OAT1 cultured in optimized FCS-free medium. Higher capacity could indicate a more mature monolayer as compared to proliferating cells, that have low reserve capacities but higher ATP-linked respiration (Aschauer, et al. 2013). Follow-up studies should also investigate the connection between the bioenergetic phenotype and proliferation. To the best of our knowledge, limited evidence is available on the comparison of bioenergetics for cells cultured in FCS-containing or hPL-containing medium. The RNA-seq data supports a more oxidative phenotype and more prominent ß-oxidation of fatty acids for FCS-free cultures. *PPARGC1A* gene expression, a marker for mitochondrial biogenesis (Bhargava and Schnellmann 2017), was upregulated (Log2FC FCS-free over FCS was 1.7). Rate-limiting enzymes for glycolysis (Lan, et al. 2016) and glucose uptake transporters were downregulated in FCS-free cultures (Fig. S6b). In addition, *PDK4*, which is associated with increased utilization of fatty acids (Pettersen, et al. 2019), was strongly upregulated and *CPT1A*, the rate-limiting enzyme for mitochondrial fatty acid import in the mitochondria (Bhargava and Schnellmann 2017), was also upregulated (Fig. S6b). Follow-up studies could look into changes in TCA metabolites as was done by Vriend et al. (Vriend, et al. 2019) for ciPTEC and compare this to primary proximal tubule cells.

A limitation of the comparison between medium conditions in this study is the data normalization step, viz*.* to total cellular protein content. This normalization step does not account for potential differences in mitochondrial content and cristae density, differences in amount and activity of enzymes participating in the metabolic pathway or in membrane potential. Therefore, more insights need to be gained into mitochondrial biology between medium conditions, *e.g.* morphological differences (mitochondrial swelling and fragmentation, disruption of mitochondrial cristae), mitochondrial mass (via *e.g.* citrate synthase activity or mtDNA copy number), mitochondrial membrane potential, fusion, fission, mitochondrial biogenesis and mitophagy (Bhargava and Schnellmann 2017). ROS and ATP production should also be studied in more detail, also considering that mRNA expression of genes related to antioxidant defenses (CAT (Bhargava and Schnellmann 2017)) was upregulated in the optimized FCS-free medium condition.

Transition to FCS-free medium for statically cultured bioengineered kidney tubules in this study was primarily focused on optimization of culture conditions to improve consistent cell coverage and to assess metabolic activity. Increasing hPL from 1 to 10% impacted metabolic activity and cell coverage mostly, suggesting that certain components in hPL are important for the maturation of ciPTEC-OAT1 on the HFM. Whether the effect of hPL was direct (*i.e.* components of hPL such as nutrients or proteins directly required for cell growth), or indirect (*e.g.* triggering alterations in expression of genes important for 3D cell culture such as extracellular matrix or cell adhesion proteins) cannot be determined in the current study. Nevertheless, it highlights that hPL should be titrated and optimized for different in vitro systems, even when using the same cell source. Extracellular matrix proteins can be important for cell attachment and differentiation, among them laminins (Matlin, et al. 2017). The specific laminins tested in this study were determined by a combination of physiological relevance (laminin-511 is the most abundant in the PT basement membrane (Miner 1999)) and RNA-seq data that showed downregulation of 2 subunits of laminin-332 in 2D-cultured ciPTEC-OAT1 cultured in optimized FCS-free medium versus FCS-containing medium (LAMB3, LAMC2, https://esbl.nhlbi.nih.gov/Databases/ciPTEC-OAT1/.). These were tested with or without collagen IV as it is known that laminins and collagen form networks within the tubular basement membrane and it has been hypothesized that a more relevant ECM provided to PTECs in vitro may help maintain their key characteristics (van Genderen, et al. 2018). Coatings did not impact general cellular metabolism nor cell coverage in this study. Rocking, to provide dynamic culture conditions, also had a positive impact on general cell metabolic activity. Follow-up studies should focus in more detail on evaluation of cell polarity, barrier integrity and transepithelial transport function of substrates for ciPTEC-OAT1 in a perfusable HFM-based system (Jansen, et al. 2016).

Finally, it is worth mentioning that in this study, the impact of medium composition was tested on an existing cell line. Future research should also address testing serum-free culture conditions for establishing new immortalized proximal tubule cell lines.

In conclusion, this study highlights significant efforts and strategies in transitioning from FCS-containing to FCS-free medium for ciPTEC-OAT1 cultures on plastic well plates and in 3D on HFM. Addition of hPL was required to support longer-term cell expansion. Culture of ciPTEC-OAT1 in optimized FCS-free medium facilitates studies that investigate mitochondrial respiration. Furthermore, the impact of medium formulations on important endpoints for a proximal tubule in vitro system to study drug transport and toxicity was highlighted. More studies are required to further characterize and recommend the culture of ciPTEC-OAT1 under these new conditions.

## Supplementary Information

Below is the link to the electronic supplementary material.

ESM 1 (DOCX 26.2 MB)

## Data Availability

Mean RNA-seq read counts data is available on https://esbl.nhlbi.nih.gov/Databases/ciPTEC-OAT1/. The proteomics dataset generated during the current study is available in the PeptideAtlas, under submission PASS05923 ([http://www.peptideatlas.org/PASS/PASS05923](http:/www.peptideatlas.org/PASS/PASS05923)). Other data described in the manuscript are available in the article itself, the supplementary materials or upon request from the corresponding author.
