# Supplementary material for: Transition to fetal calf serum-free culture enhances kidney proximal tubule cell bioenergetics and allows pharmacological applications
Source: Cell Tissue Res. 2026 May 23;404(2):13. doi: 10.1007/s00441-026-04072-7 (PMC13197463; doi:10.1007/s00441-026-04072-7)
Supplement: Supplementary file 1 — (DOCX 26.2 MB) [file 441_2026_4072_MOESM1_ESM.docx]

Transition to fetal calf serum-free culture enhances kidney proximal tubule cell bioenergetics and allows pharmacological applications

**Thomas K. van der Made^1*^, Pim Cleijpool^1^, Polly Paul^1^, Devon Barnes^1^, Stefan Oswald^2^, Bonnie Broeksma^3^, Quentin Faucher^1^, Silvia M. Mihăilă^1^, Rosalinde Masereeuw^1^**

1. Department of Pharmaceutical Sciences, Pharmacology, Utrecht University, Utrecht, The Netherlands.
2. Institute of Pharmacology and Toxicology, Rostock University Medical Center, 18057 Rostock, Germany.
3. Danone Nutricia Research, Utrecht, The Netherlands.

***Corresponding author**: Thomas K. van der Made, PhD, Utrecht University, Universiteitsweg 99, 3584 CG, Utrecht, The Netherlands [t.k.vandermade@uu.nl](mailto:t.k.vandermade@uu.nl)

**Supplemental Tables, Figures and methods**


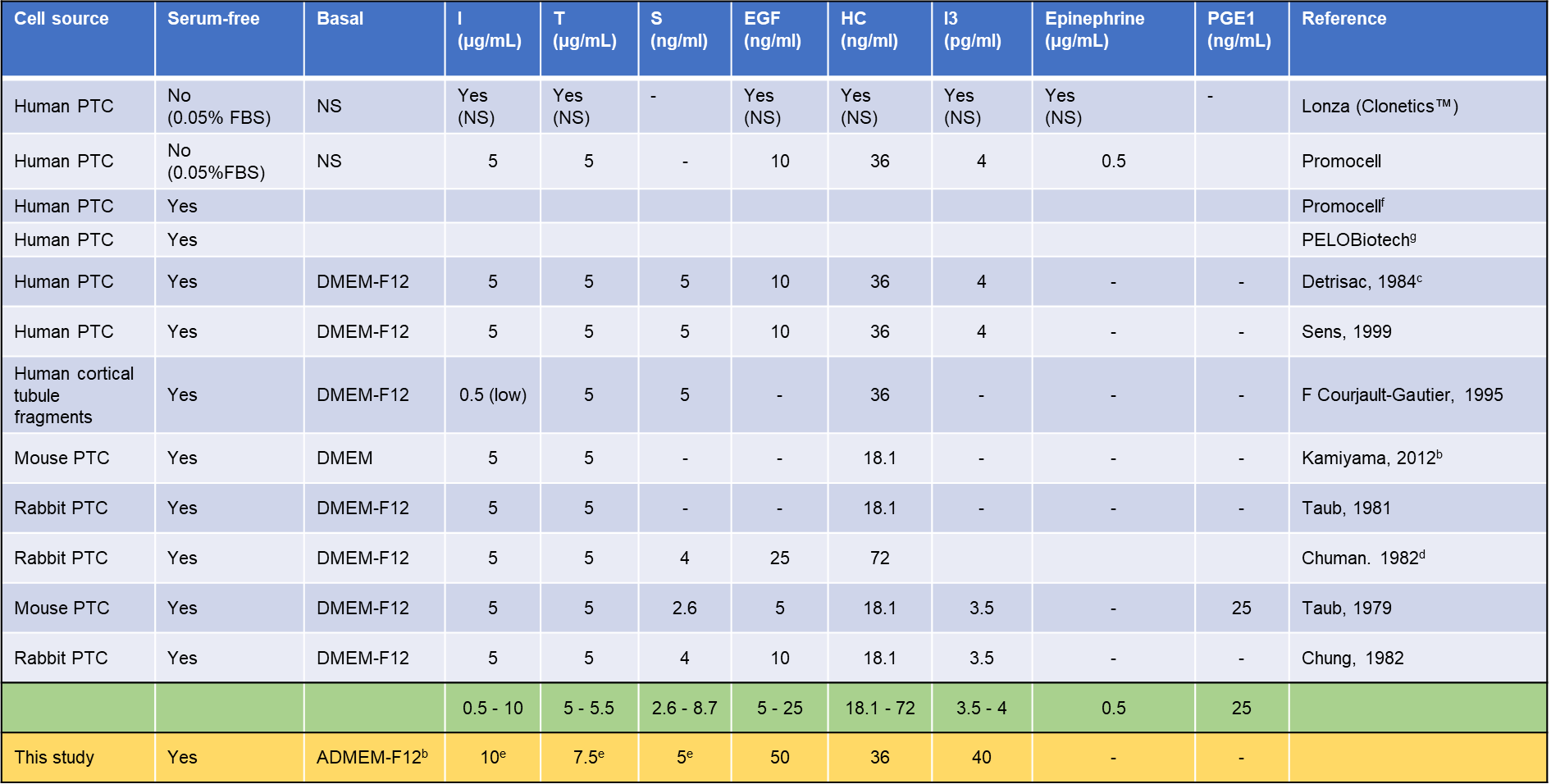
**Supplemental Table 1**. Not complete list of studies that have reported FCS-free medium formulations for culture of proximal tubule cell sources. PTC = proximal tubule cell, FBS = Fetal Bovine Serum, I = insulin, T = transferrin, S = selenium, EGF = epidermal growth factor, HC = hydrocortisone, I3 = tri-iodothyronine, PGE1 = prostaglandin E1, ADMEM-F12 = (Advanced) Dulbecco's Modified Eagle Medium/Nutrient Mixture F-12. Most studies used DMEM-F12 as basal medium supplemented with insulin, transferrin, selenium, hydrocortisone and epidermal growth factor (EGF). Several studies supplemented the medium with tri-iodothyronine and one study used the addition of prostaglandin 1. (Chuman, et al., 1982, Chung, et al., 1982, Courjault-Gautier, et al., 1995, Detrisac, et al., 1984, Kamiyama, et al., 2012, Lin, et al., 2019, Sens, et al., 1999, Taub, et al., 1979, Taub and Livingston, 1981)

^a^Ascorbic acid (3.5 µg/mL) was also added. ^b^Glutamax added.  ^c^A lot of growth supplements tested (e.g. HC, I3, PGE1, PGF2a, Progesterone, Putrescine, glucagon, aldosterone, testosterone, dihydrotestosterone, estradiol, kallikrein, calcium, l-glutamine, l-cysteine, FGF, EGF, PTH). ^d^Lysine vasopressin (100 µU/mL) was also added. ^e^Already present in ADMEM. ^f^<https://www.avantorsciences.com/us/en/product/14612873/renal-epithelial-cell-growth-medium-promocell>. ^g^https://www.pelobiotech.com/product-details/renal-epithelial-cell-growth-medium-kit-defined.html?_gl=1*1atzd78*_up*MQ..*_gs*MQ..*_ga*MTA4ODU1ODI5OS4xNzUxMjc3NTMw*_ga_PCKJ6LN2DP*czE3NTEyNzc1MjkkbzEkZzEkdDE3NTEyNzc1NDUkajQ0JGwwJGgw&gclid=Cj0KCQjw64jDBhDXARIsABkk8J73JzZMIjJh6SOSIxb7KYoOsKzvxclCgJC8KPZrAc4uCXBF7XBnHUIaAj30EALw_wcB&gbraid=0AAAAACE4L04rbdYtRN_2exN_ekjEtSScy

| **Top 25 genes most down- or up-regulated based on -log10 adjusted p value** | | | | |
| --- | --- | --- | --- | --- |
| **Down** | Log2FC | adjPval |  |  |
| COL6A3 | -2.7 | 0 | Found to be upregulated in focal segmental glomerulosclerosis samples | (Bai, et al., 2022) |
| GALNT15 | -3.0 | 2.9e^-270^ | No literature in relation to *in vitro* proximal tubule cells experiments found |  |
| PDK1 | -2.7 | 1.3e^-146^ | Plays an important role in cellular responses to hypoxia and is important for cell proliferation under hypoxia. | (Schley, et al., 2012) |
| APLN | -4.0 | 1.7e^-135^ | No literature in relation to *in vitro* proximal tubule cells experiments found |  |
| PPFIA4 | -4.4 | 1.5e^-123^ | Signal transduction, upregulated by HIF-1α | (Wang, et al., 2005) |
| BNIP3 | -2.8 | 1.7e^-120^ | Involved with apoptosis, upregulated by HIF-1α | (Wang, et al., 2005) |
| BNIP3P1 | -2.9 | 4.2e^-106^ | Involved with apoptosis, upregulated by HIF-1α |  |
| SLC37A2 | -2.9 | 3.4e^-104^ | No literature in relation to *in vitro* proximal tubule cells experiments found |  |
| FOXQ1 | -3.0 | 4.3e^-108^ | No literature in relation to *in vitro* proximal tubule cells experiments found |  |
| LUM | -2.3 | 1.6e^-107^ | No literature in relation to *in vitro* proximal tubule cells experiments found |  |
| AK4 | -1.2 | 9.4e^-108^ | No literature in relation to *in vitro* proximal tubule cells experiments found |  |
| HK2 | -2.4 | 1.1e^-102^ | Rate-limiting enzymes for glycolysis increased in IRI | (Lan, et al., 2016) |
| PLOD2 | -1.5 | 9.7e^-101^ | PLOD2 is upregulated in renal cell carcinoma | (Cao, et al., 2021) |
| FAM20C | -2.6 | 2.5e^-88^ | No literature in relation to *in vitro* proximal tubule cells experiments found |  |
| C1QL1 | -1.9 | 2.4e^-91^ | No literature in relation to *in vitro* proximal tubule cells experiments found |  |
| DUXAP9 | -1.0 | 5.2e^-87^ | Overexpression of DUXAP9 promoted renal cancer cells proliferation and epithelial to mesenchymal transition | (Tan, et al., 2021) |
| FN1 | -1.5 | 1.0e^-84^ | Found to be upregulated in focal segmental glomerulosclerosis samples | (Bai, et al., 2022) |
| SH3D21 | -1.7 | 1.1e^-85^ | No literature in relation to *in vitro* proximal tubule cells experiments found |  |
|  |  |  |  |  |
| **Up** |  |  |  |  |
| PSAT1 | 1.4 | 1.0e^-144^ | No literature in relation to *in vitro* proximal tubule cells experiments found |  |
| PLEKHA1 | 1.3 | 7.6e^-138^ | Upregulated PLEKHA1 induced OXPHOS in proximal tubule cells, overexpression caused oxidative stress in kidneys |  |
| SOD3 | 4.0 | 3.2e^-125^ | Gene related to antioxidant defense |  |
| SPP1 | 1.9 | 1.3e^-104^ | Proximal tubule injury marker |  |
| JAG1 | 2.6 | 5.9e^-90^ | TGFβ1 induces JAG1 upregulation | (Nyhan, et al., 2010) |
| NHSL2 | 2.1 | 9.4e^-86^ | No literature in relation to *in vitro* proximal tubule cells experiments found |  |
| PINCR | 1.9 | 1.4e^-87^ | No literature in relation to *in vitro* proximal tubule cells experiments found |  |

**Supplemental Table 2.** Identified **Top 25** most down- or up-regulated genes in FCS-free ciPTEC-OAT1 cultures based on -log10 adjusted p-value and potential relation to the proximal tubule cell *in vitro*.

**Supplemental Table 3**. Quantitative information on individual Seahorse outcome parameters. Mean ± SD (%CV) is shown of data highlighted in Fig.5. Values were generated using Seahorse Analytics software and GraphPad prism to combine experiments and compute mean, SD and %CV.

|  | **FCS-containing medium** | | **Optimized FCS-free medium** | | |
| --- | --- | --- | --- | --- | --- |
| Seeding Density | 6000 cells/well | 12000 cells/well | 6000 cells/well | 12000 cells/well | 18000 cells/well |
| Basal respiration (pmol/min/µg protein) | 2.2 ± 0.9 **(41.8%)** | 3.1 ± 1.2 **(37.4%)** | 5.1 ± 1.9 **(37.8%)** | 4.3 ± 1.4 **(33.6%)** | 4.7 ± 1.8 **(38.6%)** |
| Maximal Respiration (pmol/min/µg protein) | 3.5 ± 1.3 **(37.4%)** | 4.4 ± 1.5 **(34.1%)** | 21.6 ± 7.3 **(33.9%)** | 22.0 ± 7.3 **(33.4%)** | 25.4 ± 9.8 **(38.7%)** |
| Spare Respiratory Capacity (pmol/min/µg protein) | 1.3 ± 0.6 **(47.3%)** | 1.3 ± 0.8 **(64.7%)** | 16.5 ± 5.5 **(33.4%)** | 17.6 ± 5.9 **(33.6%)** | 20.7 ± 8.0 **(38.9%)** |
| ATP-production coupled Respiration (pmol/min/µg protein) | 1.5 ± 0.7 **(45.9%)** | 2.3 ± 0.9 **(37.7%)** | 3.6 ± 1.3 **(36.4%)** | 3.2 ± 0.9 **(29.2%)** | 3.6 ± 1.3 **(36.3%)** |
| Proton Leak (pmol/min/µg protein) | 0.7 ± 0.3 **(39.8%)** | 0.9 ± 0.3 **(40.9%)** | 1.5 ± 0.9 **(60.5%)** | 1.1 ± 0.7 **(61.6%)** | 1.0 ± 0.6 **(57.8%)** |
| Non-mitochondrial Oxygen Consumption (pmol/min/µg protein) | 1.2 ± 0.8 **(63.8%)** | 1.4 ± 0.4 **(31.4%)** | 3.1 ± 1.2 **(38.4%)** | 2.8 ± 1.2 **(44.3%)** | 3.1 ± 1.1 **(34.0%)** |
| Protein (µg/well) | 8.3 ± 2.6 **(30.9%)** | 8.8 ± 2.4 **(26.9%)** | 5.6 ± 1.5 **(27.6%)** | 7.1 ± 2.0 **(28.8%)** | 7.4 ± 2.2 **(30.4%)** |
| No. of independent experiments | 3 | 3 | 3 | 3 | 3 |
| No. of datapoints | 33 | 32 | 33 | 33 | 34 |

FCS 6 x 10^3^ cells/well 12 x 10^3^ cells/well


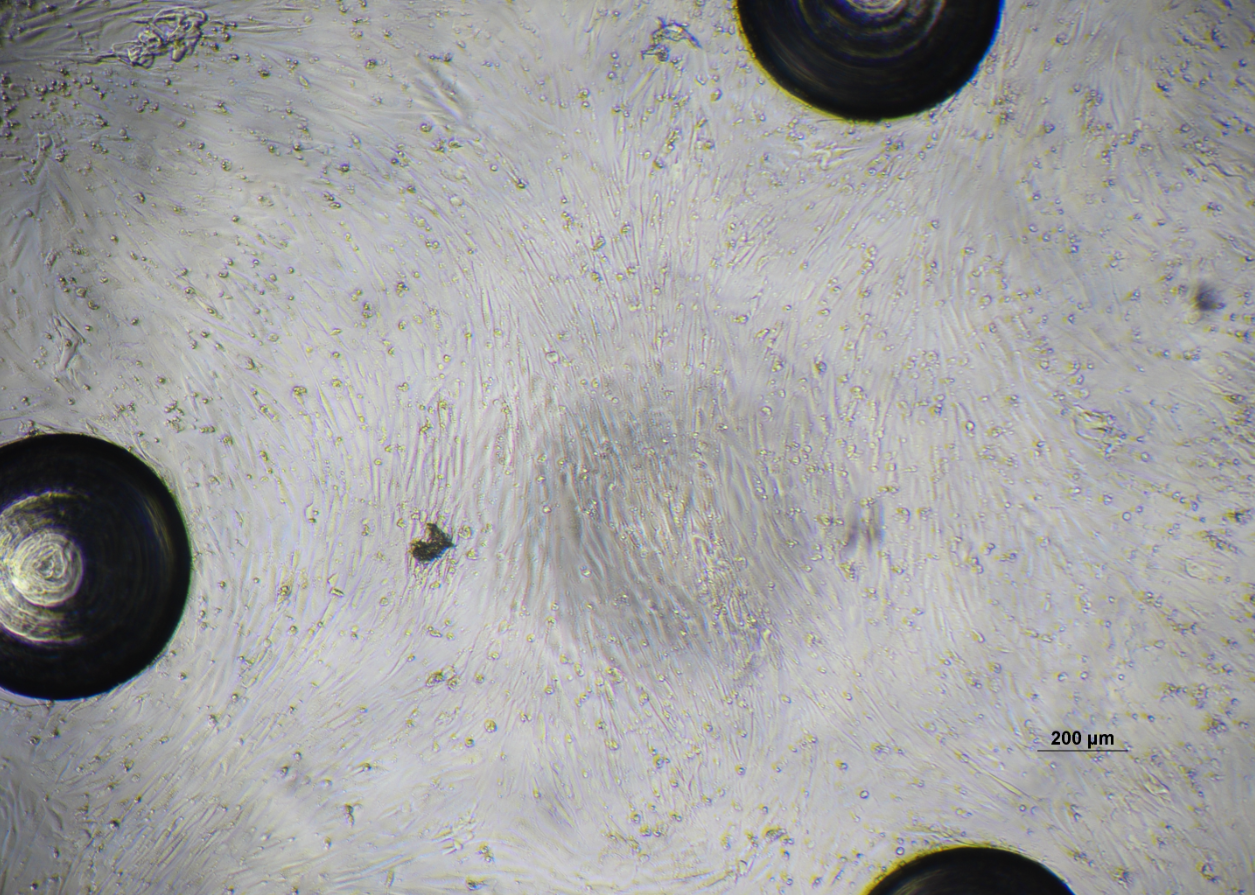

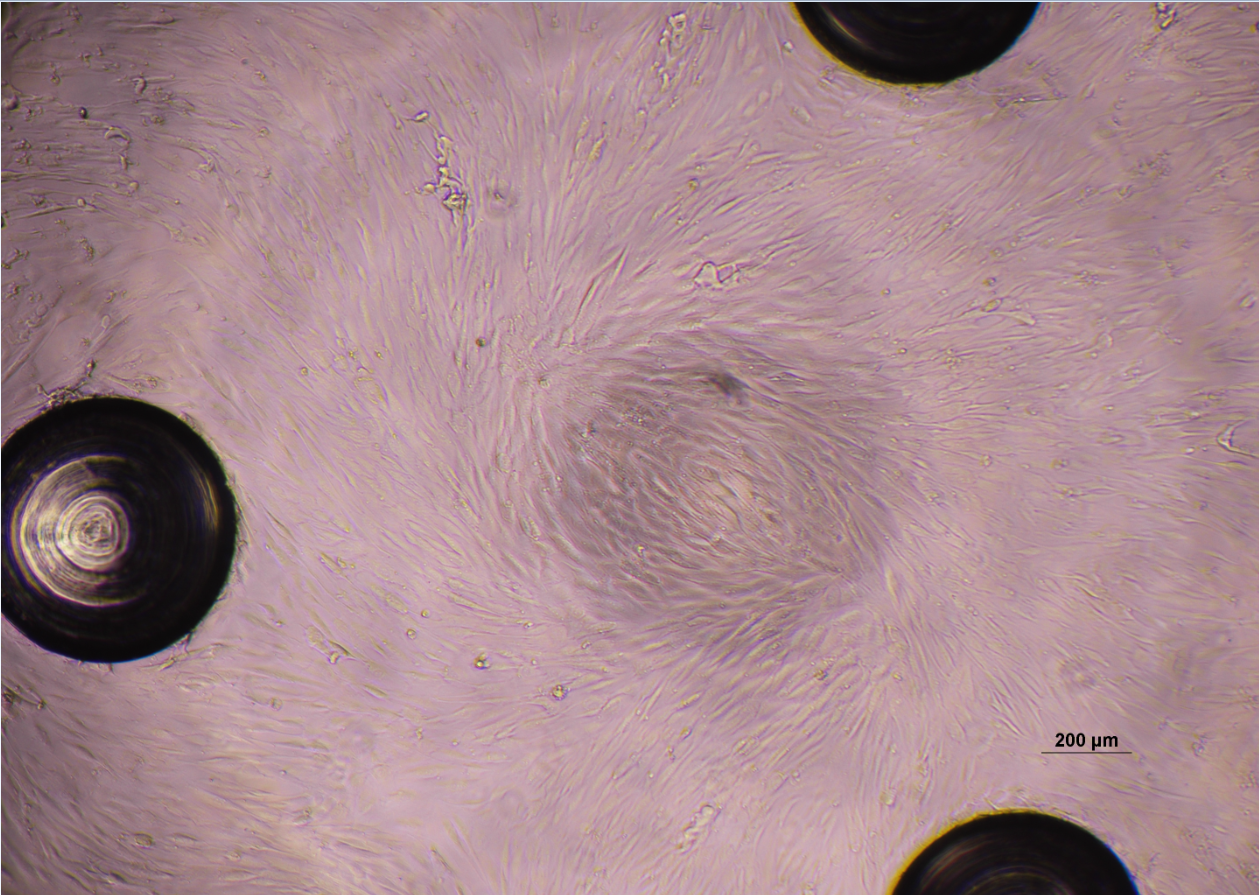

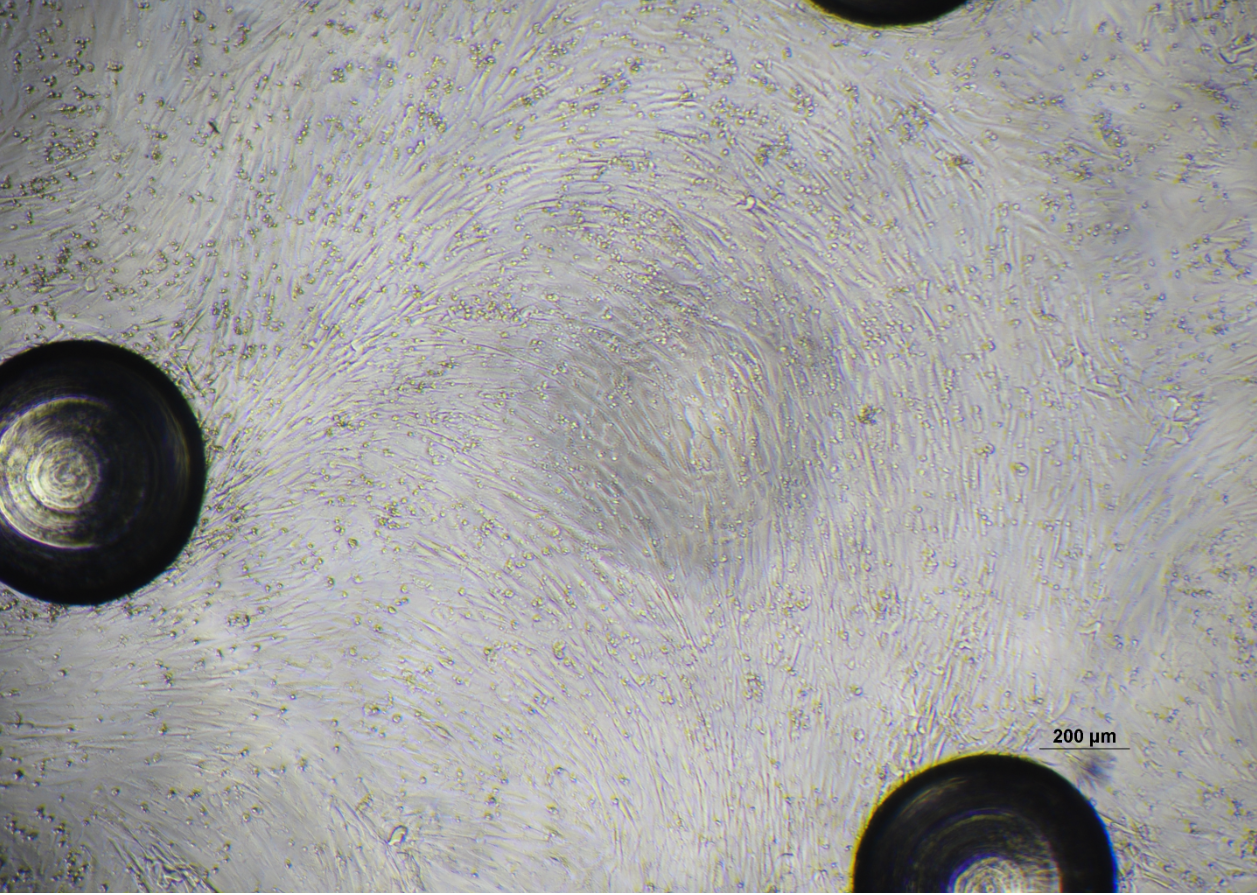

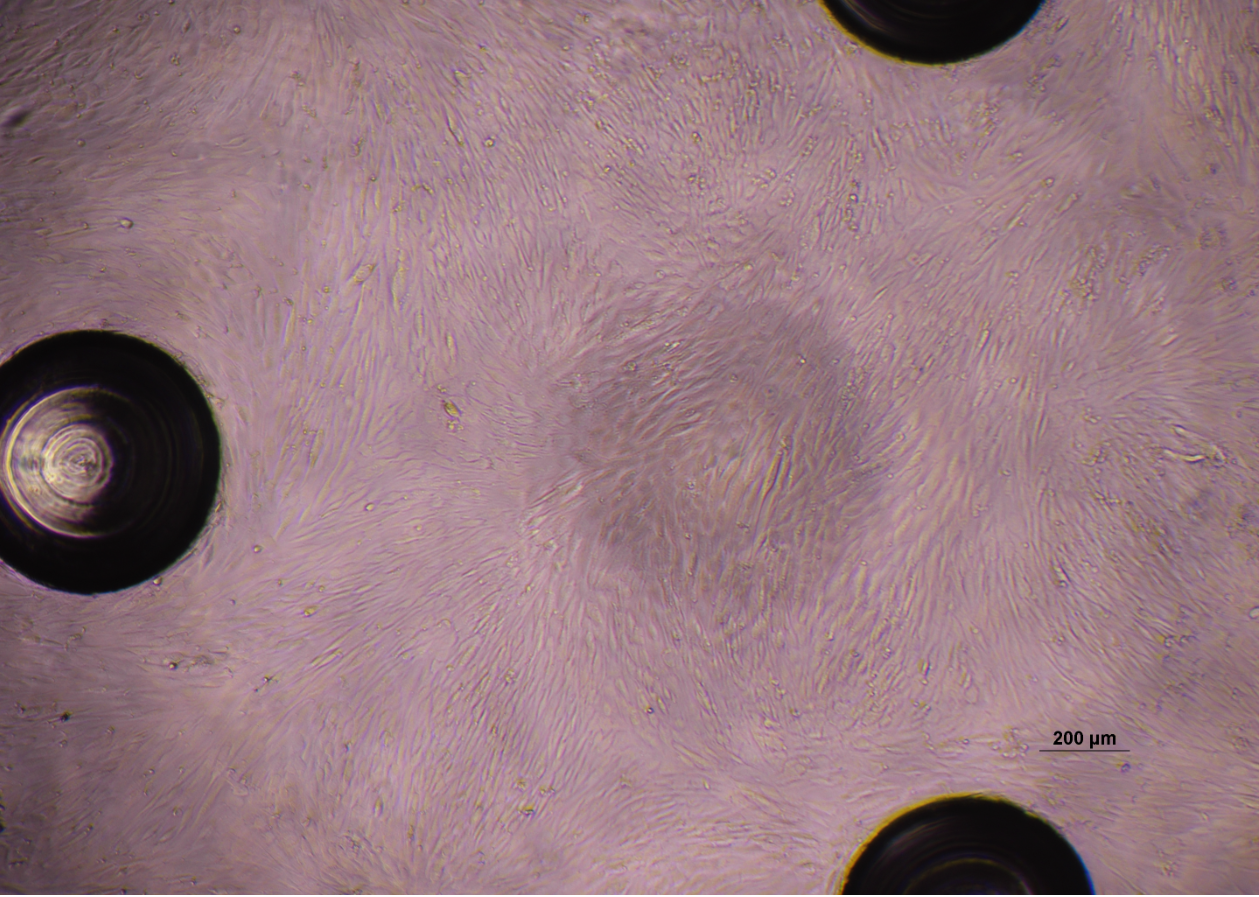

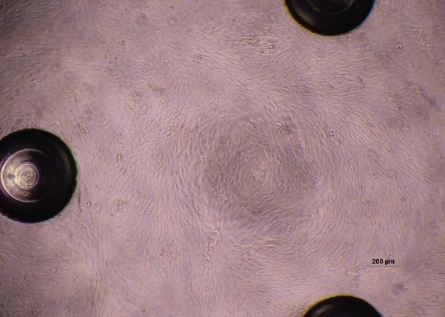


FCS-free, 6 x 10^3^ cells/well 12 x 10^3^ cells/well

FCS-free, 18 x 10^3^ cells/well

**Supplemental Figure 1.** Representative brightfield microscopic image of the different conditions one day prior to the Seahorse assay.

**
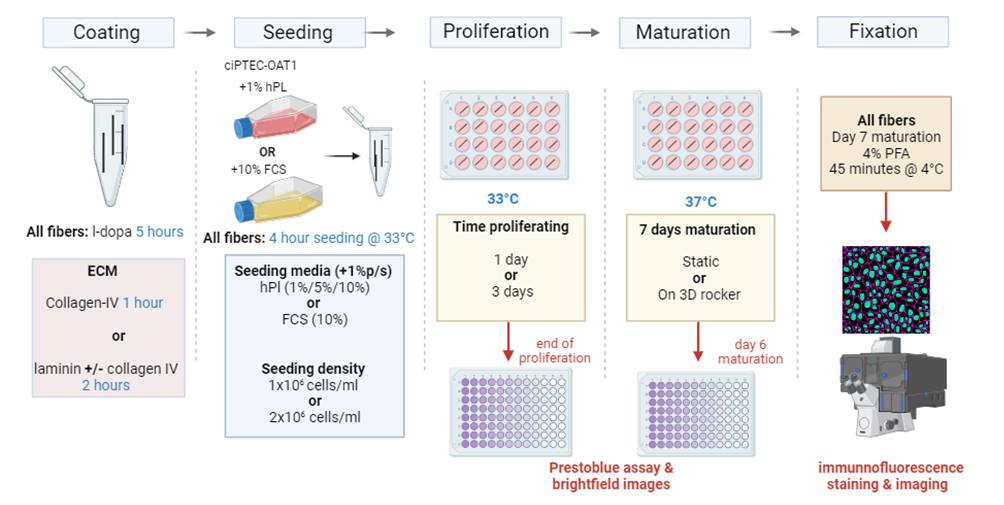
**

**Supplemental Figure 2.** Schematic overview of optimization of HFM conditions.


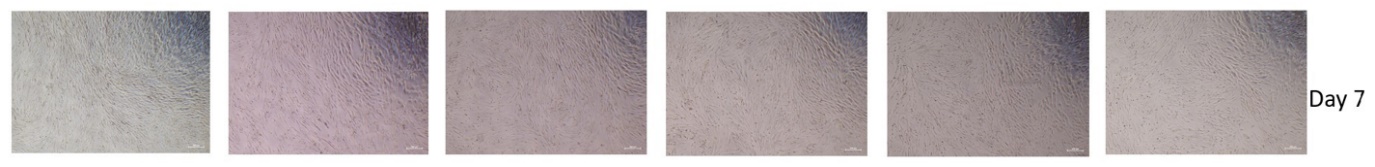

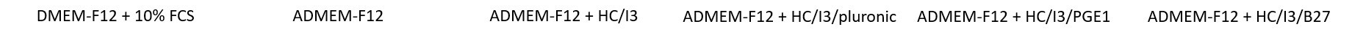


**Supplemental Figure 3**. Representative brightfield microscopic image per media formulation condition prior to the PrestoBlue experiment in 24-well plates.


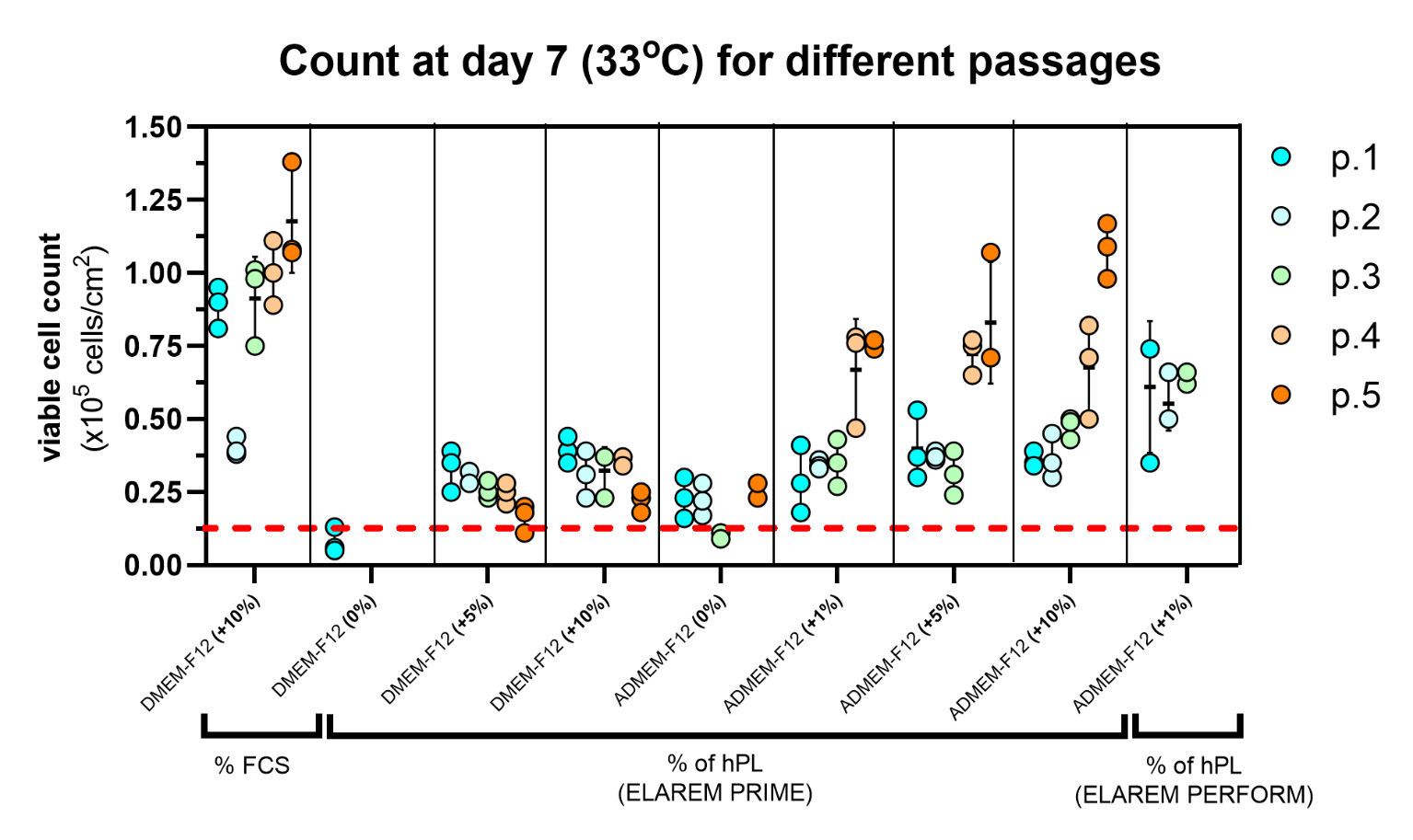


**Supplemental Figure 4. Evaluation of longer-term ciPTEC-OAT1 culture after culture in FCS-containing or various FCS-free medium conditions.** Number of viable cells per cm^2^ at day 7 for multiple passages (p.#) after transition to FCS-free cell culture (1,5 or 10% ELAREM Prime^TM^, 1% ELAREM Perform^TM^ and using DMEM-F12 or ADMEM-F12) or the FCS control. An initial seeding of 0.12 x 10^5^ viable cells/cm^2^ was used (red dashed line). Depicted datapoints are from 1 independent experiment performed in triplicates. Data for passage 4 for ADMEM-F12 (0%) was excluded due to a technical error, one datapoint for ADMEM-F12 (+0%) and ADMEM (+1% ELAREM PRIME) for passage 5 was excluded due to infection. Counts for passage 4 and 5 in ADMEM-F12 + 1% ELAREM Perform^TM^ were not measured. DMEM-F12 (0%) was not measured after passage 1 due to limited amount of cells in the wells. ciPTEC-OAT1 could not (DMEM-F12) or minimally (ADMEM-F12) be maintained in FCS-free medium formulations without hPL formulations. Addition of ELAREM Prime^TM^ or ELAREM Perform^TM^ to ADMEM-F12 led to an increase in cell coverage and cell counts, especially for passage 4 and 5 for ELAREM Prime^TM^. Addition of ELAREM Prime^TM^ to DMEM-F12 as direct replacement to FCS did yield more cells than initially seeded, however, cell counts were decreased as compared to ADMEM-F12 and FCS control.


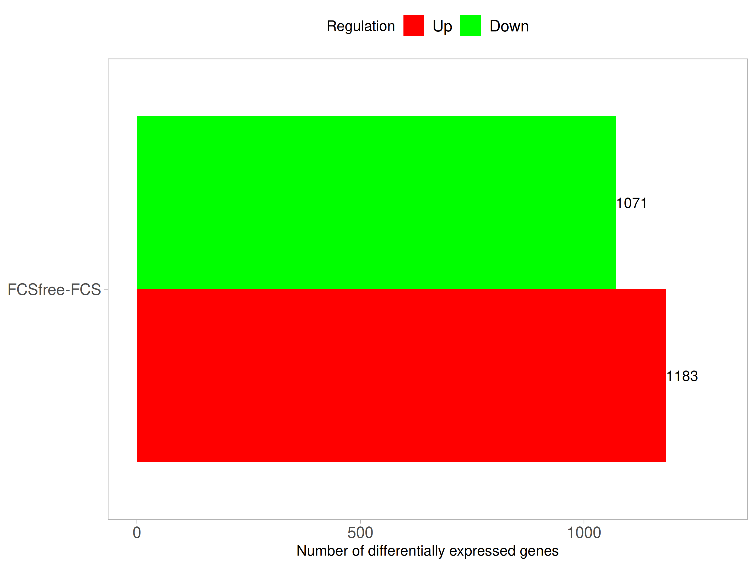

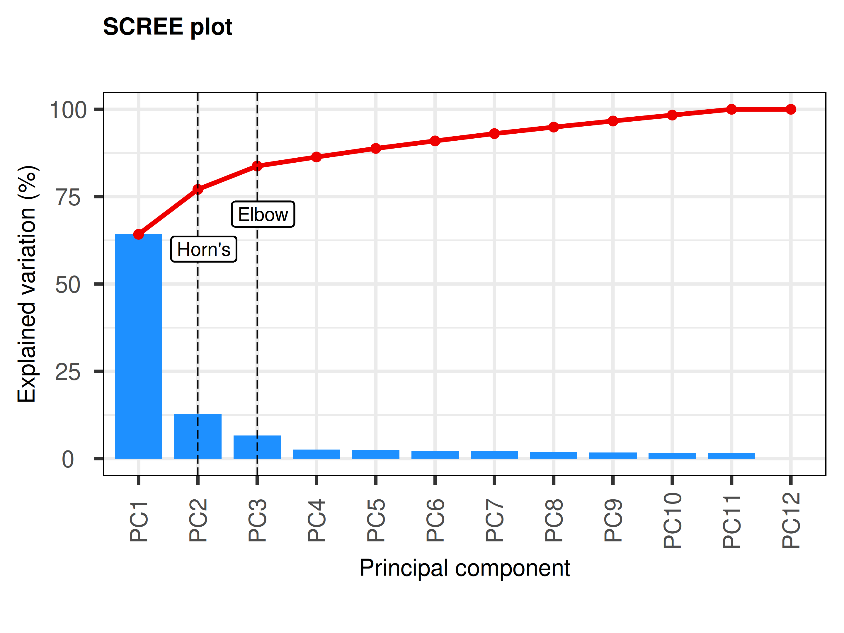
A B


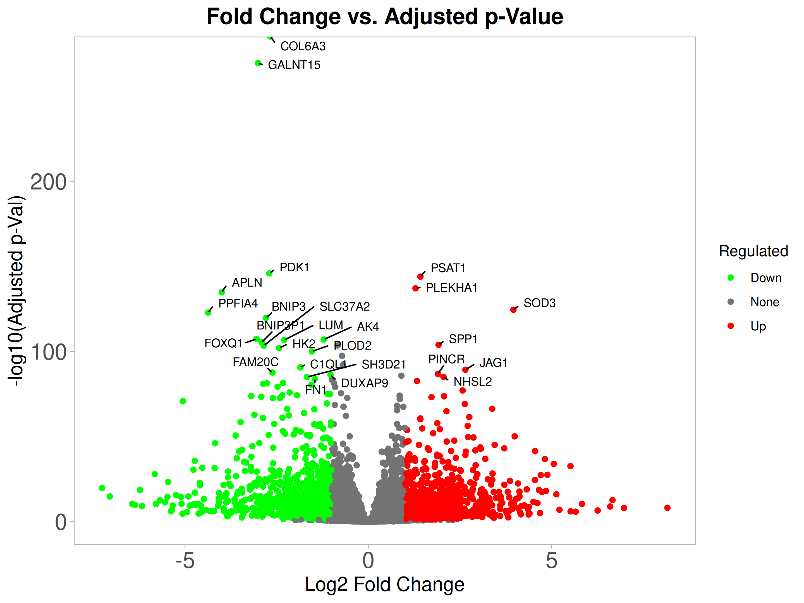

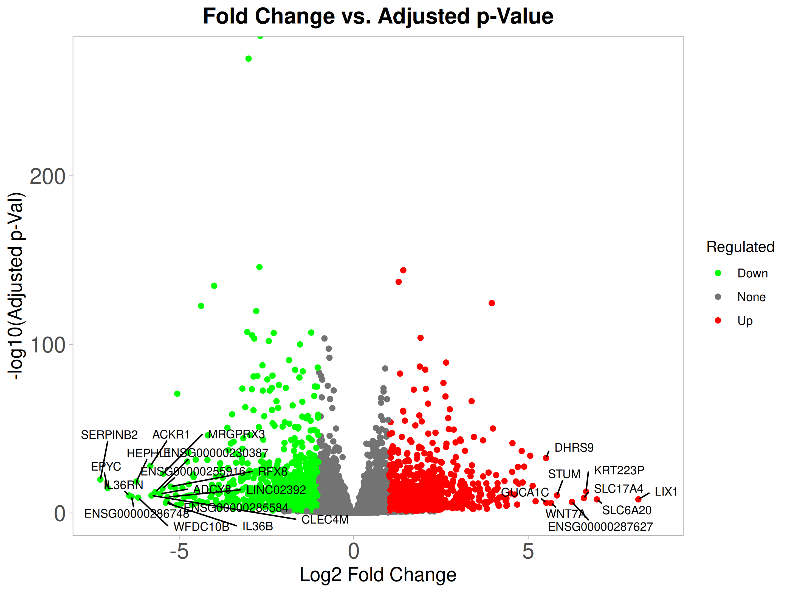
C

D


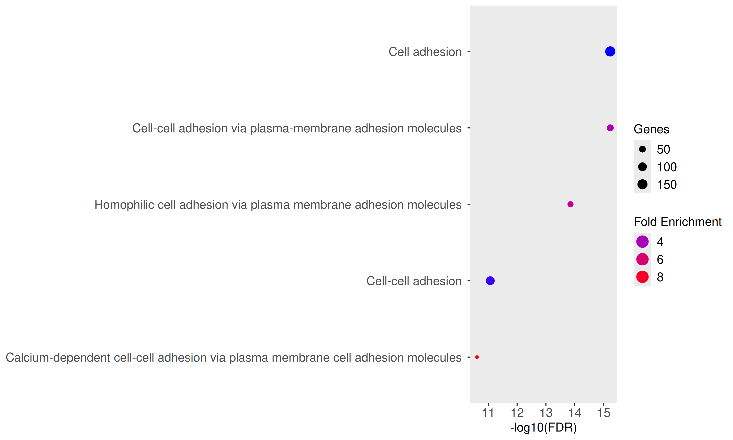


**D1** GO:BP - Upregulated


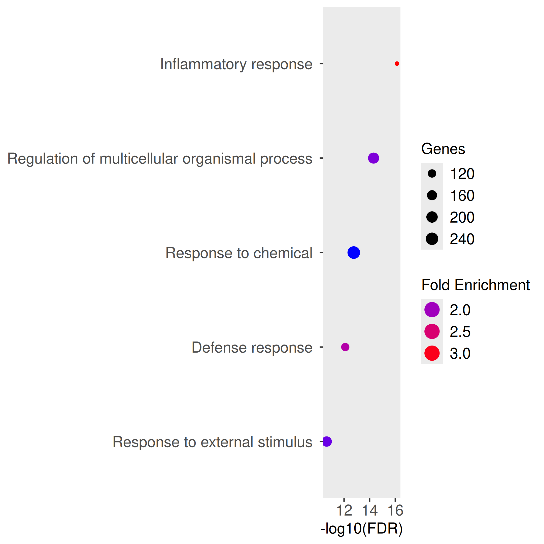


**D2** GO:BP - Downregulated


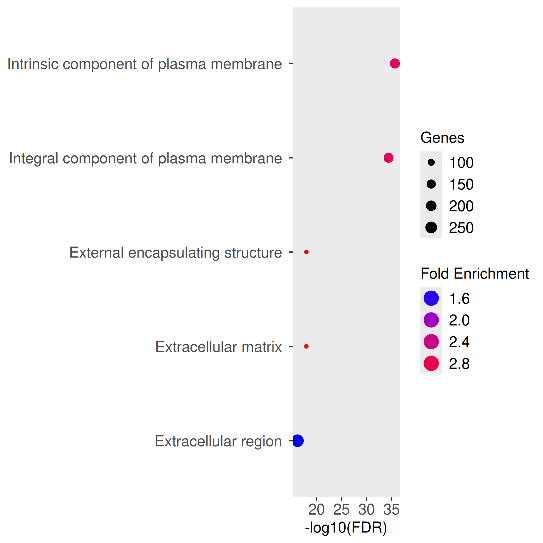


**D3** GO:CC - Upregulated


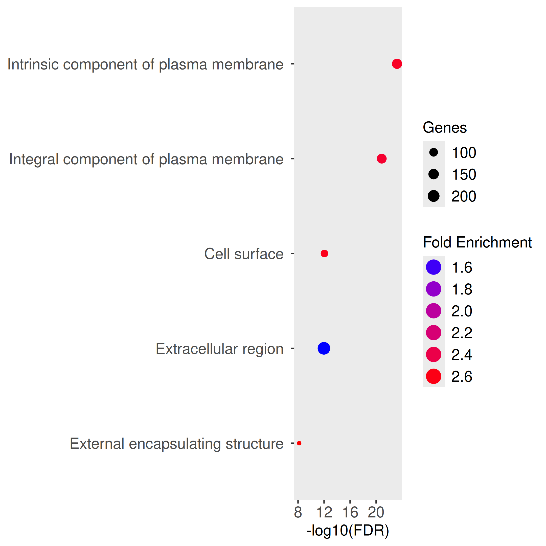


**D4** GO:CC - Downregulated


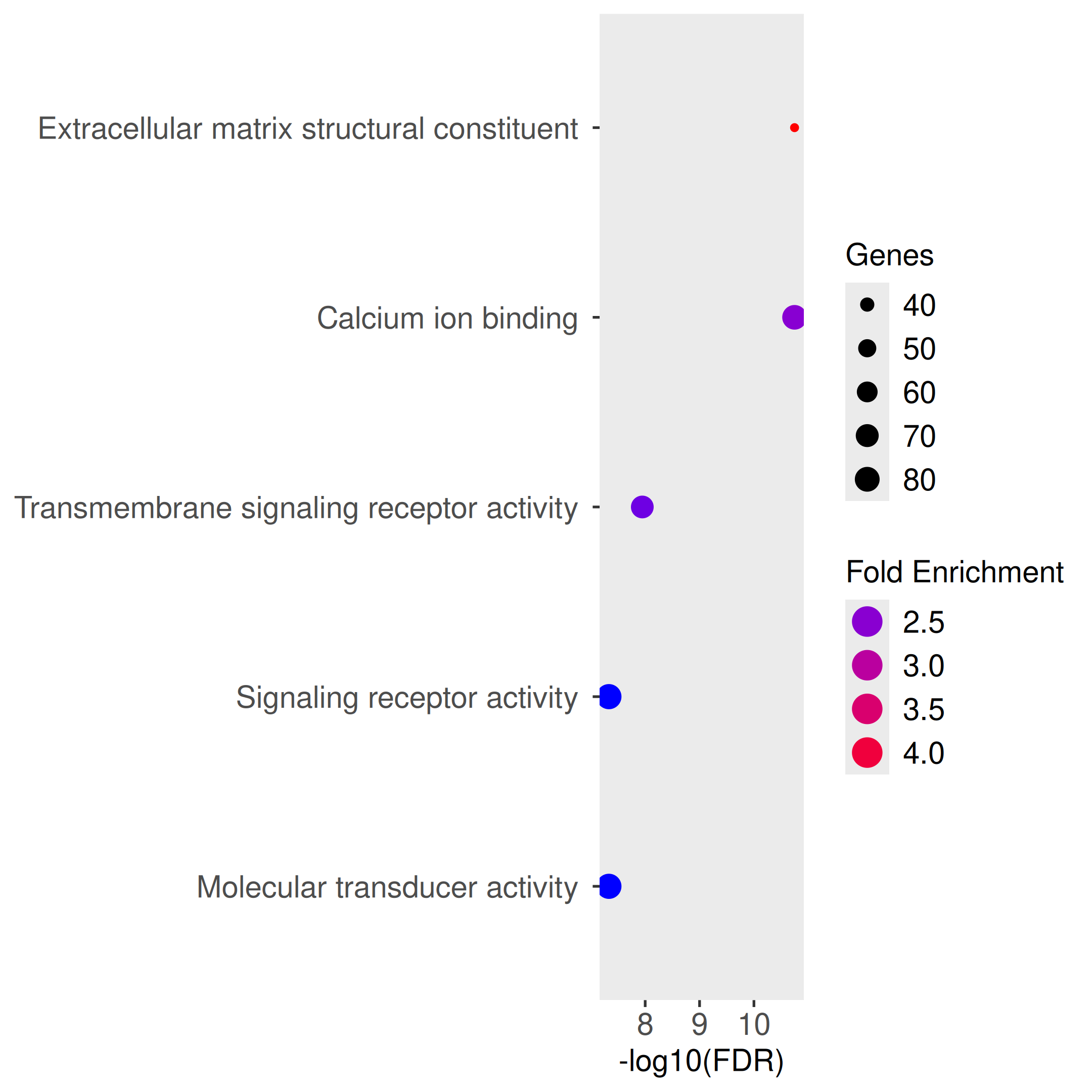


**D5** GO:MF - Upregulated


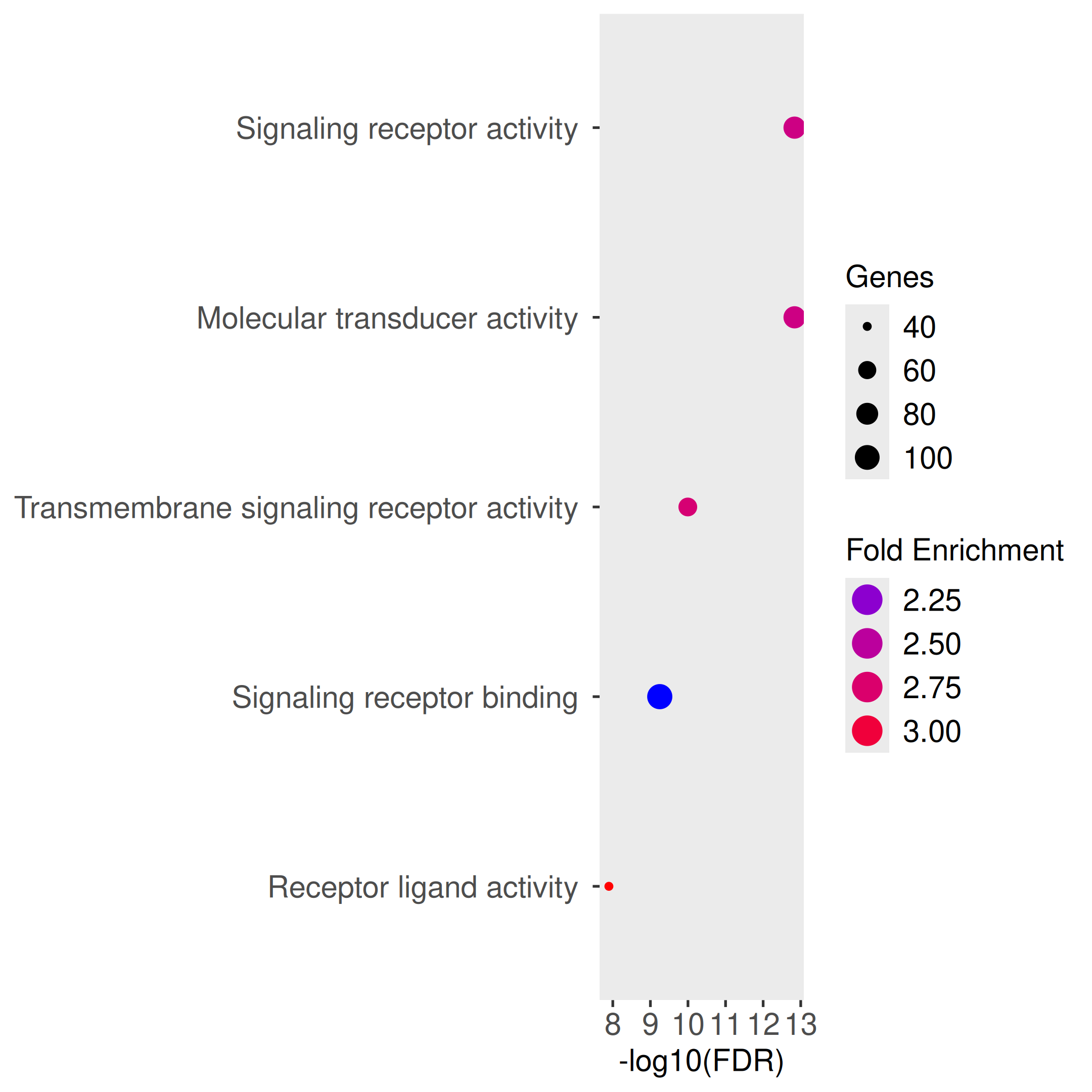


**D6** GO:MF - Downregulated

**Supplemental Figure 5**. Genome wide transcriptome analysis and transcriptomics differences between FCS-containing and optimized FCS-free cultures. **(a)** Scree plot for clustering of ciPTEC-OAT1 samples expanded in optimized FCS-free medium for 1 or 5 passages or their respective FCS-control. **(b)** Number of differentially expressed up- and down-regulated genes, after samples for passage 1 and 5 have been combined for the medium conditions (c) Volcano plot of up- and down-regulated genes for pooled (by passage number) FCS-free and FCS-containing ciPTEC-OAT1 samples: FDR cutoff 0.05, Min Fold Change 2. Top 25 differentially expressed genes based on Log2 Fold Change (left) or -log10 Adjusted p-value (right) are highlighted. **(d)** Up- and downregulated pathways in FCS-free compared to FCS-containing cultures for Gene Ontogeny terms (BP = biological process; CC = cellular component; MF = molecular function) is shown. 3 replicates per condition have been inputted individually in iDEP 1.13 (see Supplemental Methods).


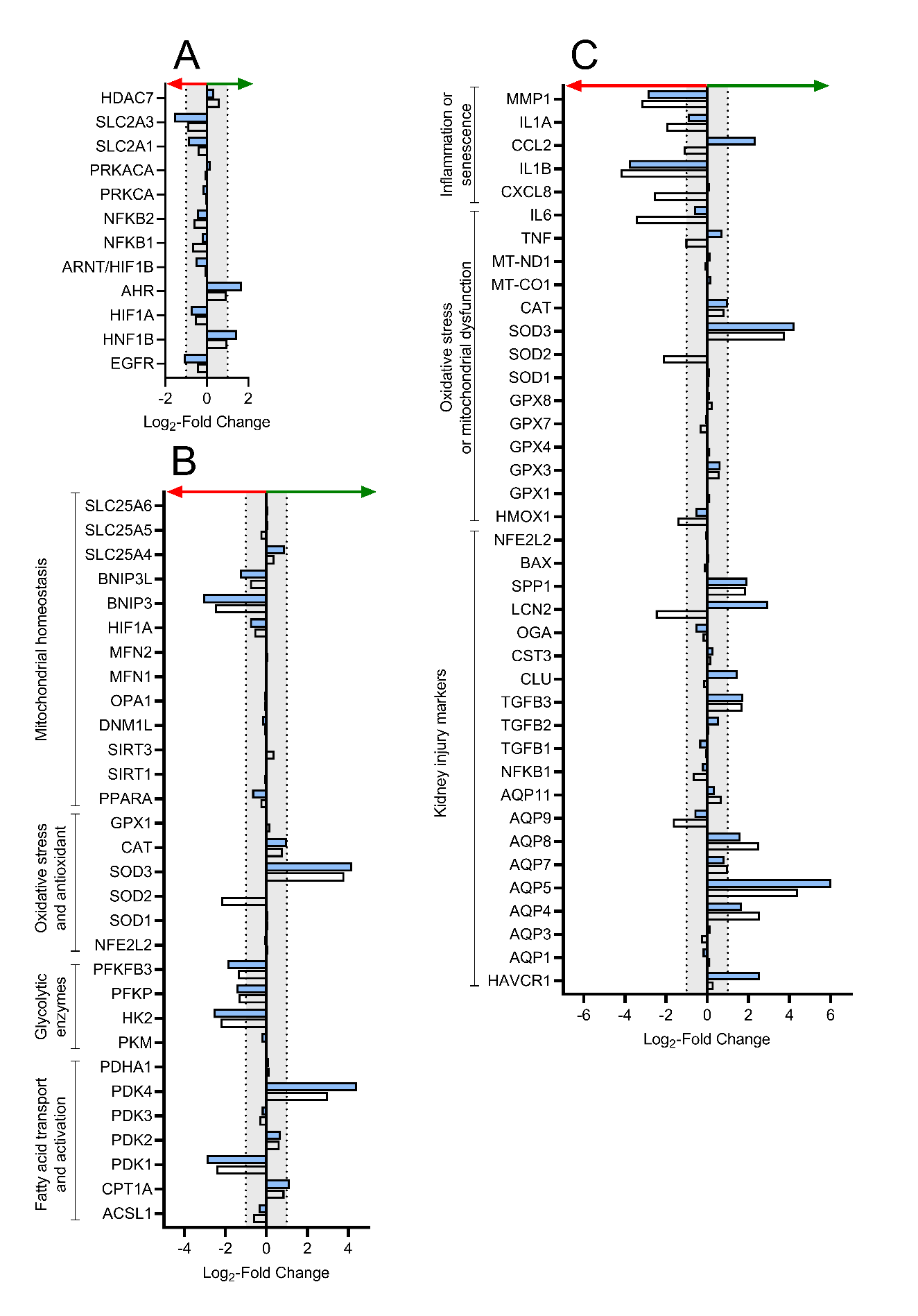


**Supplemental Figure 6.** RNAseq data for genes associated with direct or indirect OAT(1) regulation (Caetano-Pinto and Stahl, 2023) (A), mitochondrial activity (B) and more general kidney injury markers (C). White bar is FCS-fee (1 passage on FCS-free medium) versus FCS-containing ciPTEC-OAT1 culture. Blue bar is FCS-fee (5 passages on FCS-free medium) versus FCS-containing ciPTEC-OAT1 culture. Grey area highlights the within 2-fold difference. Red and green means down- and up-regulated in FCS-free medium, respectively. Selection of genes displayed was informed by literature(Barnes, et al., 2024, Bhargava and Schnellmann, 2017, Hoogstraten, et al., 2023, Lan, et al., 2016, Pettersen, et al., 2019, Shao, et al., 2024, Yang, et al., 2022).

**
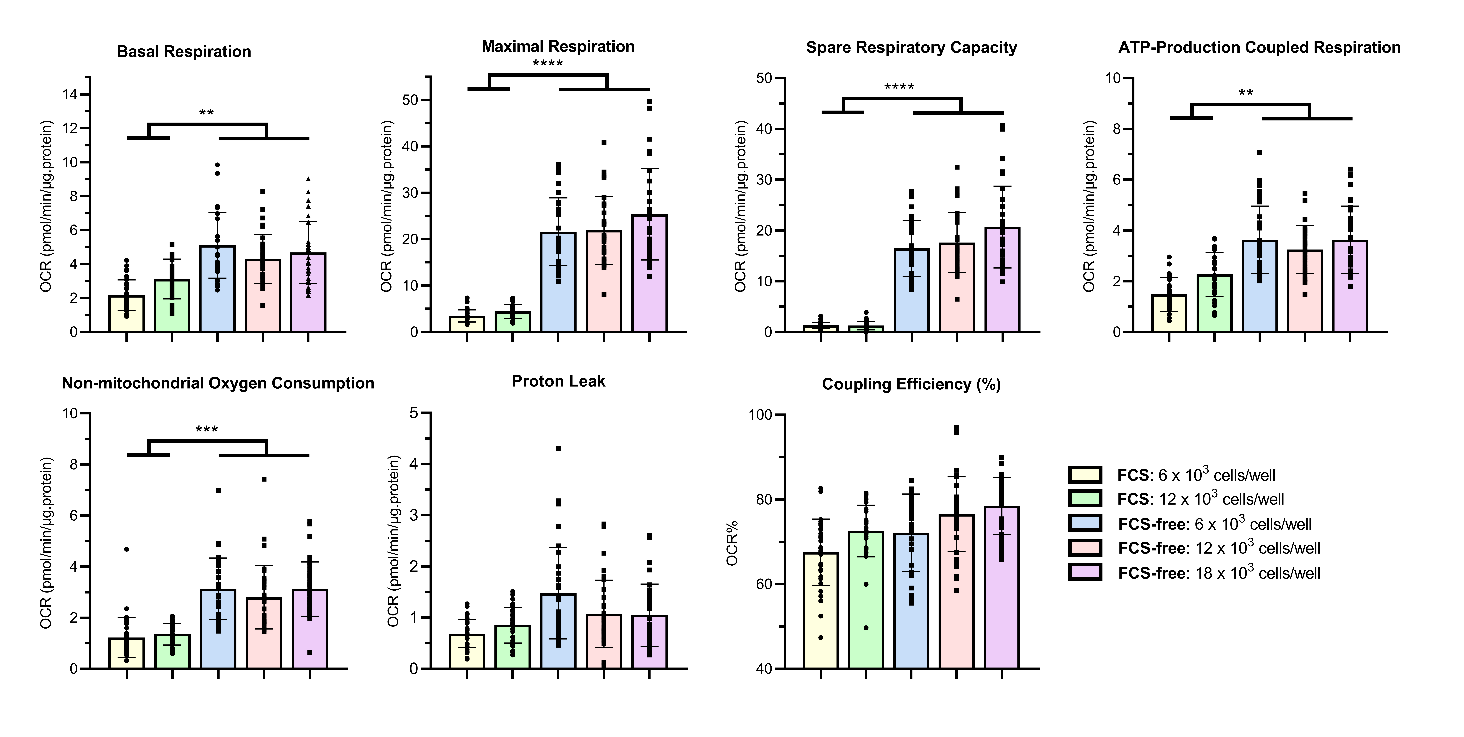
**

**Supplemental Figure 7.** Individual distribution of the datapoints for the Seahorse assay outcome parameters. Data is shown for FCS-containing: 6 x 10^3^ cells/well (■), 12 x 10^3^ cells/well (■) and FCS-free: 6 x 10^3^ cells/well (■), 12 x 10^3^ cells/well (■) and 18 x 10^3^ cells/well (■). Mean ± SD from 3 independent studies is depicted. 15 values were excluded in total based on issues with Oligiomycin, FCCP, Rotenone + Antimycin A injection and/or ECAR measurements. Two-way ANOVA was performed, comparison between FCS and FCS-free conditions highlighted, differences within cell seeding densities groups were not statistically significant. **p< .01; *** p <.001; **** p < .0001)


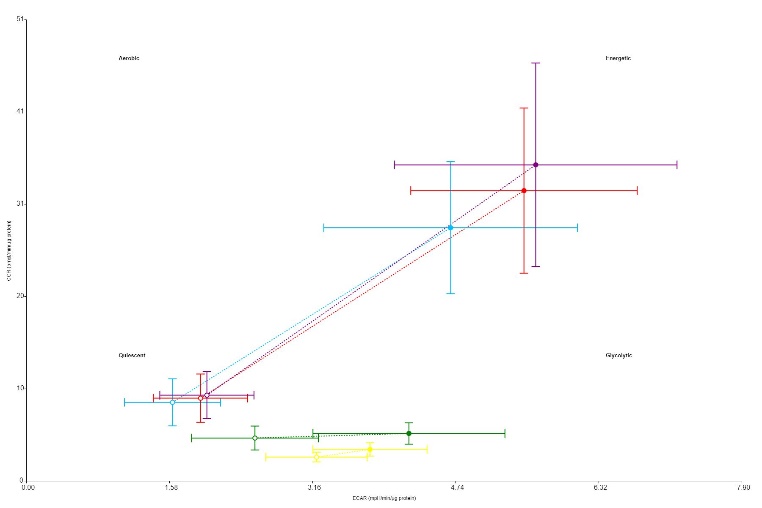

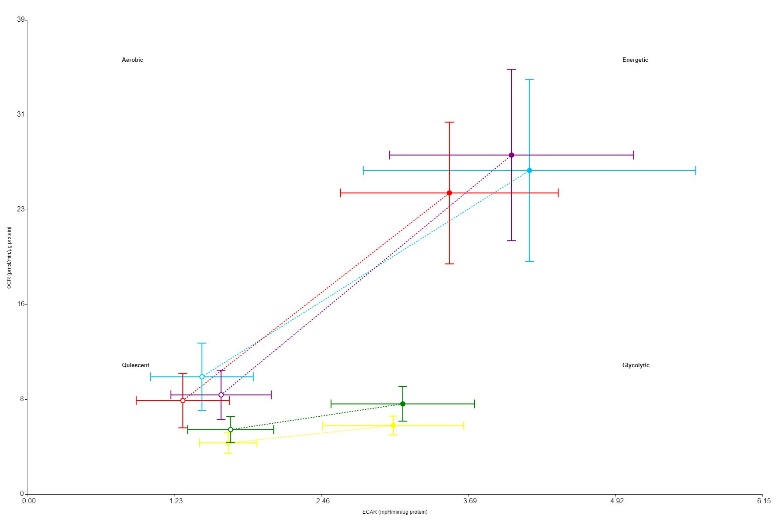

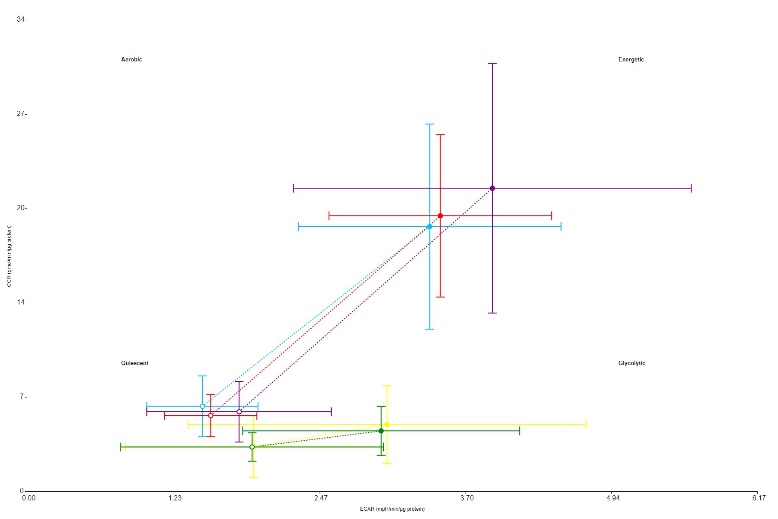

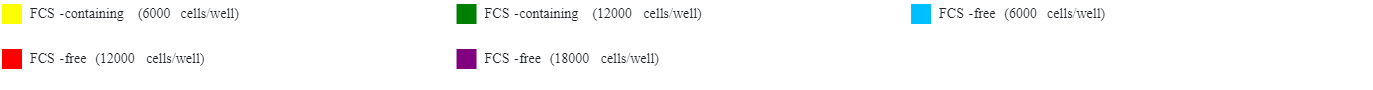


**Supplemental Figure 8.** The bioenergetic profile of the cells by plotting the OCR versus ECAR measurements. The baseline phenotype is plotted as the open marker, the stressed phenotype is filled marker, and the connecting line is metabolic potential. The XF Cell Energy Phenotype widget in Seahorse Analytics software was used, with no adjustments to visualization of the data. The plot for 3 independent experiments are highlighted.


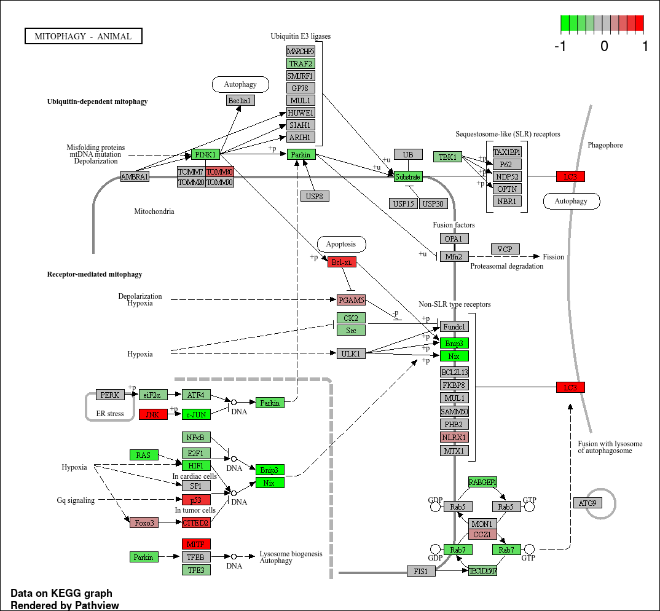

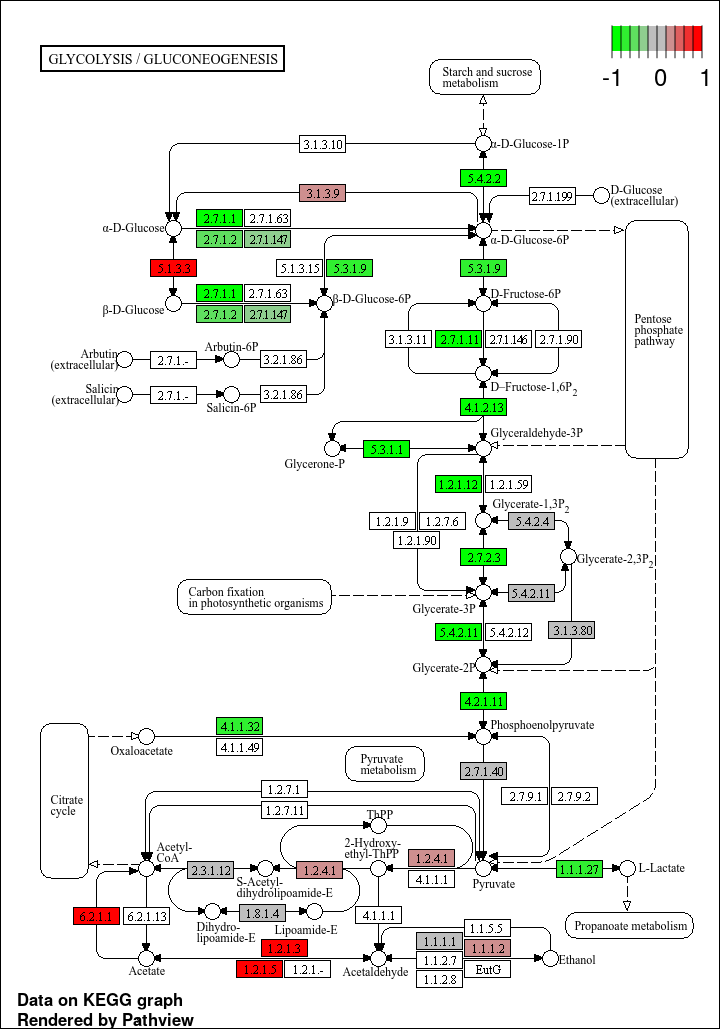

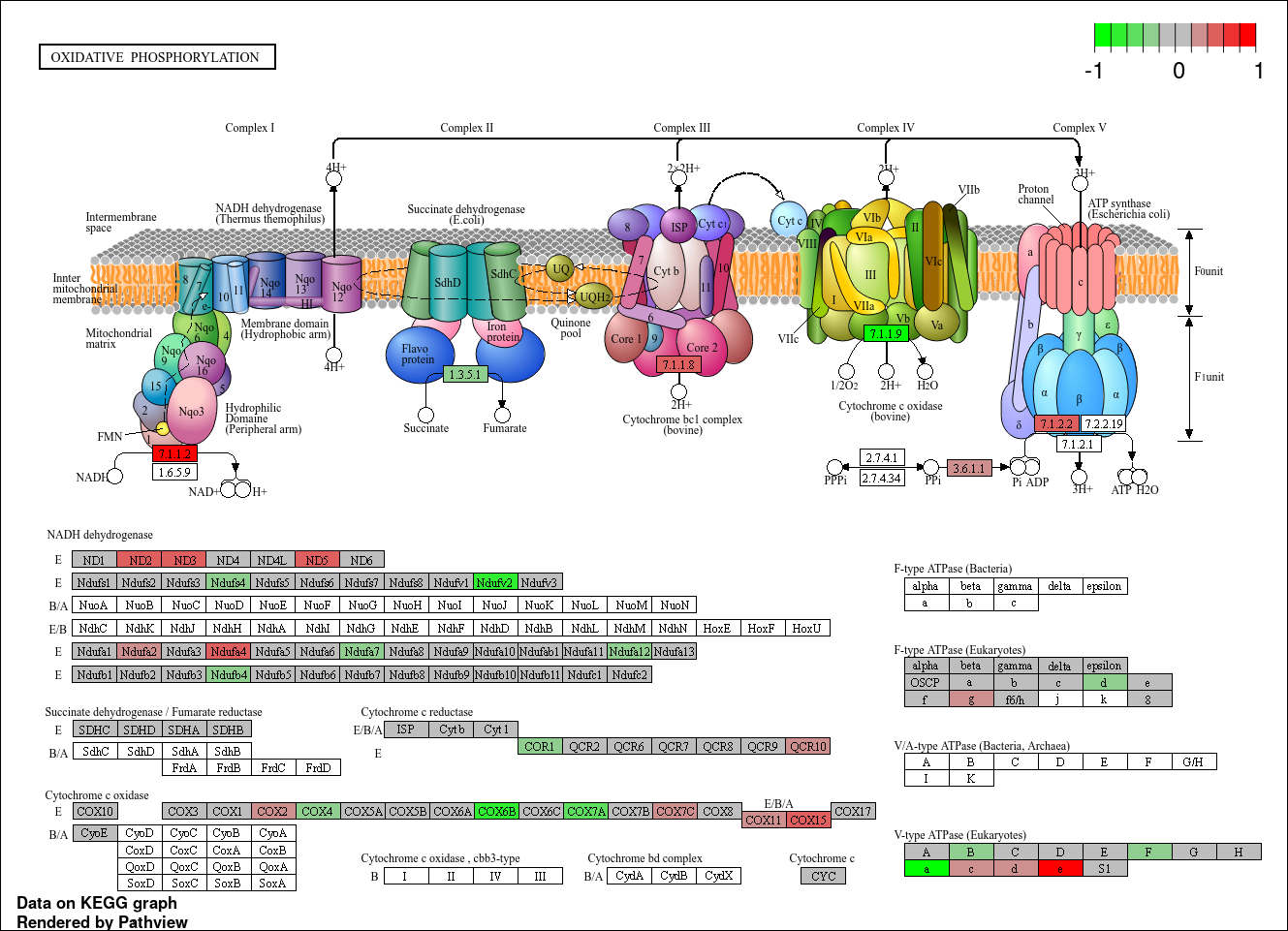


**Supplemental Figure 9.** Differences in mRNA expression of genes related KEGG pathways (Kanehisa, 2019, Kanehisa, et al., 2025, Kanehisa and Goto, 2000) for Glycolysis/gluconeogenesis (map00010), Oxidative phosphorylation (hsa00190) and Mitophagy - animal (hsa04137) for optimized FCS-free cultures versus FCS containing. Green = decreased expression in optimized FCS-free cultures, red = increased expression in optimized FCS-free cultures. Generated using iDEP 1.13 software.

**Supplemental methods**

**24-well plate coating procedure**

Coated 24-well plates were prepared as follows: 24-well plates were incubated with 1 mL sterile 3,4-Dihydroxy-L-phenylalanine (L-DOPA) solution (2 mg/mL in 10 mM TRIS buffer, pH 8.5, left to dissolve for 45 min at 37 ^o^C, swirling every 10 min) for 5h at 37 ^o^C. Subsequently, L-DOPA solution was aspirated and wells were washed 2x with HBSS. Stock solution of Collagen Type IV (Advanced Biomatrix, Carlsbad, US) was prepared according to manufacturer’s recommendation and further diluted in HBSS prior to surface coating. 1 mL of Collagen Type IV solution (25 µg/mL in HBSS) was added to the wells and plates were incubated for 1h at RT. Then, plates were washed 2x with HBSS and were ready to be used for cell seeding. Another coating that was tested, Cellnest (Fujifilm Irvine Scientific, Tilburg, The Netherlands, stock solution (0.5%)) was prepared according to manufacturer’s recommendation and was further diluted in HBSS. 24-well plates were coated with a final concentration of 20 μg/cm^2^ Cellnest solution for 1h at 37^o^C. Solution was aspirated and plates were ready to be seeded.

**Preparation of samples for transporter protein abundance determination**

First, protein in lysate samples was quantified by BCA. All centrifugation steps were performed with Vivacon® 500, 10,000 MWCO Hydrosart units (Sartorius, VN01H01, VNCT01) at 14000g at RT. Filters were wet with 1% formic acid (100 µL/well, centrifuged for 15 min). Subsequently, 100 µg sample to 200 µL with UT buffer (8 M urea, 2 M thiourea in aqua dest) was prepared and samples were centrifuged for 30 min followed by centrifugation with 200 µL urea buffer (8 M urea, 100 mM TRIS in aqua dest., pH 8.5) for 30 min. Collection tubes were emptied and samples were incubated with 100 µL DTT (8 mM Dithiothreitol in urea buffer) at 56 °C for 15 min. Subsequently, samples were centrifuged for 25 min. Thereafter, 100 µL urea buffer was added and samples were centrifuged for 25 min and were incubated with 100 µL IAA (50 mM Iodacetamid in urea buffer) thereafter at RT in the dark for 20 min. Samples were centrifuged for 25 min, then centrifuged with 100 µL urea buffer for 25 min and the collection tube was emptied. Furthermore, samples were incubated with 100 µL DTT at 56 °C for 15 min, then centrifuged for 25 min, subsequently centrifuged with 100 µL urea buffer for 25 min followed by centrifugation (twice) with 100 µL ABC ((65 mM Ammoniumbicarbonate in aqua dest., pH 7.8)) for 25 min. Filter unit was transferred to a new collection tube and incubated with 100 µL trypsin (Promega, V5113) solution containing 1% Protease Max®-solution (Promega, V2071) at 37 °C in a wet chamber for 16 h. The next day, 10 µl of 10% formic acid was added and centrifuged for 10 min at RT followed by centrifugation with 40 µL ABC for 10 min. Final eluate was stored at -80 °C until analysis.

**Evaluation of ciPTEC-OAT1 transcriptomics in optimized FCS-free and FCS-containing cultures**

For isolation, the “miRNA CT 400” protocol was run on a QIAsymphony isolation robot using the QIAsymphony RNA Kit (931636) on each sample in 400 µl of the RLT Plus lysis buffer (1053393). For library preparation, RNA quality was checked with the Agilent Fragment Analyzer 5300 system using the RNA Kit (15nt) (Cat. DNF-471-1000). RNA quantity was measured with the Invitrogen™ Qubit ™ Fluorometer using the Qubit RNAa HS Assay Kit (Cat. Q32855). 100ng of total RNA was used to prepare TruSeq Stranded mRNA libraries (Cat. 20020594) following the manufacturers protocol, with custom 384 xGen UDI-UMI adapters from IDT. After the library preparation libraries were checked with the Fragment Analyzer system dsDNA 910 Reagent Kit (35-1500bp) (Cat. DNF-910-K1000) and with Qubit dsDNA HS Assay Kit (Cat. Q32854). Sample libraries were pooled equimolar. Libraries were sequenced on a Nextseq2000 (Illumina) by using a P3 flowcell with 50bp paired-end reads. Quality control on the sequence reads from the raw FASTQ files was done with FastQC (v0.11.8). TrimGalore (v0.6.5) as used to trim reads based on quality and adapter presence after which FastQC was again used to check the resulting quality. rRNA reads were filtered out using SortMeRNA (v4.3.3) after which the resulting reads were aligned to the reference genome fasta (GCA_000001405.15_GRCh38_no_alt_plus_hs38d1_analysis_set) using the STAR (v2.7.3a) aligner. Followup QC on the mapped (bam) files was done using Sambamba (v0.7.0), RSeQC (v3.0.1) and PreSeq (v2.0.3). Readcounts were then generated using the Subread FeatureCounts module (v2.0.0) with the Homo_sapiens.GRCh38.106 gtf file as annotation.

**Analysis of read counts using publicly available iDEP1.13 software for determining DEGs and enrichment pathways**: Read counts data of 3 replicates (seeded from the same biological pool, and subsequently cultured independently for 1 day at 33 ^o^C and 7 days at 37 ^o^C) for 1 passage on FCS-free medium and the 3 replicates for 5 passages on FCS-free medium were combined and the same was done for FCS control cultures. Subsequently, 6 replicates for FCS condition and 6 replicates for FCS-free condition were inputted in iDEP1.13 in 3. Expression data in the Load Data tab (see screenshot below). It is therefore important to note that there are 2 strong biological replicates and for every strong biological replicate 3 weaker ones (from the same biological pool but matured independently, in different wells).


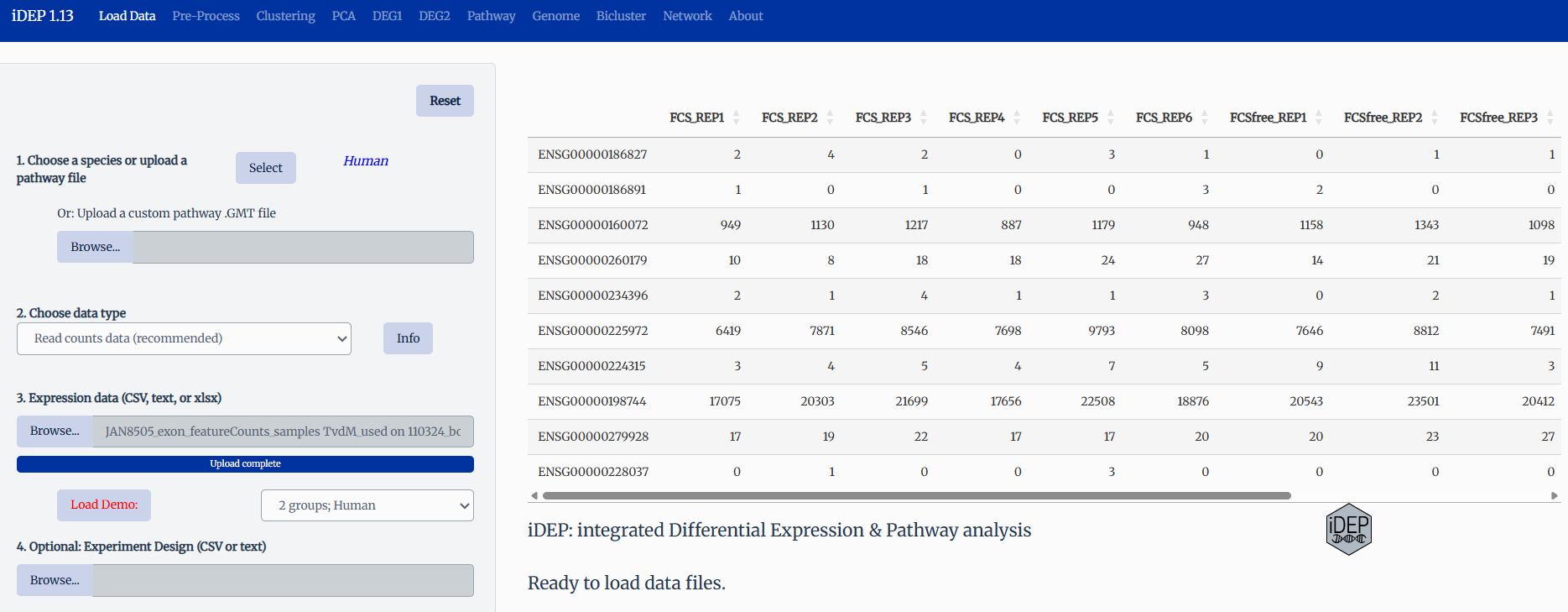


In the pre-process stage, the software (1) filter out genes with extremely low expression, (2) Gene IDs are converted to Ensembl gene ID or STRING-db gene ID, and (3) apply transformations. In the Pre-Process tab in iDEP1.13 Min. CPM was set to 0.5 in n libraries of 2. Transform counts data for clustering & PCA was set to “EdgeR:log2(CPM+c)”, pseudo count c to “4” and missing values to “Use gene median”. More detailed explanations can be found in the info section of the Pre-Process tab at iDEP1.13. This led to 19148 genes (out of 61551 genes in 12 samples) passing the filter. 19131 were converted to Ensembl gene IDs. The remaining 17 genes were kept in the data using original IDs. Next, in the DEG1 tab “DESeq2” was filled for the method, with a FDR cutoff of “0.05” and min fold-change of “2”, ticking the box of “independent filtering of lower counts”. On the right hand side, the box for FCSfree vs. FCS was ticked to select comparisons among sample groups. The used R code is available on the website. The volcano plots and enrichments pathway plots in Supplemental Figure 5 were generated using the DEG2 tab in iDEP1.13. Further details on the enrichment analysis are available when you select Enrichment and then details in the DEG2 tab. KEGG pathways shown in Supplemental Figure 9 were generated under the Pathway tab. For comparison, “FCSfree-FCS” was inputted, the pathway analysis method was: “GSEA (preranked fgsea)” and the pathways database was “KEGG”. The pathway significance cutoff (FDR) was put to “0.05”. These pathway analyses are done using fold-changes values of all genes calculated by DESeq2, more information is available in the info tab on the website.

# **Evaluation of cellular bioenergetics by Seahorse- XF cell mitostress test**

The oxygen consumption rate (OCR) and extracellular acidification rate (ECAR) were monitored in real-time using the Seahorse extracellular flux analyzer XF 96 (Agilent, Technologies, Amstelveen, The Netherlands). Prior to cell seeding, XF96-well microplates (ref # 103793-100) were coated with 50 µL Collagen Type IV solution (25 µg/mL in HBSS) at RT for 1h, covered in aluminum foil. ciPTEC-OAT1 cells were seeded at various cell seeding densities in FCS-containing (6 or 12 x 10^3^ cells/well) or FCS-free + 1% ELAREM^TM^ Perform (6, 12 or 18 x 10^3^ cells/well) medium using a randomized plate lay-out. ciPTEC-OAT1 cultures were passaged for at least one passage in FCS-free medium prior to seeding. Cells were seeded in 80 µL final volume, the outer wells of the plate were filled with HBSS only, except for the four corners of the plate which were filled with medium only to serve as background correction wells. The plate was left at RT in the tissue culture hood for an hour to promote even cell distribution. During incubation, the plate was placed towards the back of the incubator to ensure minimal environmental fluctuations. ciPTEC-OAT1 were incubated at 33°C for 1 day, and at 37°C for 7 days prior to the assay. One day prior to the assay, each well of the utility plate was filled with 200 µL of sterile MQ, the hydrobooster was placed on top and subsequently the sensor cartridge was placed. Then, the plate was placed in a humidified non-CO_2_ 37^o^C incubator overnight. In addition, XF Calibrant was aliquoted and placed in a humidified non-CO_2_ 37^o^C incubator overnight. One hour before the assay, sterile MQ was removed, 200 µL of pre-warmed XF Calibrant was added to each well and the sensor cartridge with utility plate was left in a non-CO_2_ 37^o^C incubator for approximately 60 min. Before the assay, culture medium was partially removed (leaving 40 µL). Subsequently, cells were washed twice with 160 µL of pre-warmed XF assay medium (supplemented with 2 mM L-Glutamine, 1 mM Pyruvate and 10 mM Glucose, all purchased from Agilent Technologies), leaving 40 µL after each wash. Finally, 140 μl XF assay medium was added to each well for a final volume of 180 μl/well and the cell culture plate was left in a non-CO_2_ 37°C incubator for 60 min. Compounds from a pouch of the Seahorse XF Cell Mito Stress Test Kit were unpacked and added to the hydrated sensor cartridge in the following final concentrations (in XF assay medium): Oligomycin (1.5 µM), FCCP (2.0 µM) and Rotenone/Antimycin A (0.5 µM). Oligomycin and Rotenone/Antimycin concentrations were selected according to Agilent Seahorse XF Cell Mito Stress guidelines. In contrast, FCCP concentration was based on a previously performed titration experiment for ciPTEC14.4 (Faria, et al., 2023). The sensor cartridges were loaded into the Seahorse Analyzer and calibration was performed. Subsequently, after successful calibration, the utility plate was removed, the cells loaded plate inserted and the program (Wave software version 2.6.8.3) was started. After the run, the cells were lysed in 50 µL of RIPA (ThermoFisher Scientific, Amsterdam, The Netherlands) buffer for subsequent protein analysis by BCA.

**ciPTEC-OAT1 maturation on hollow fiber membranes**

The following changes were made for experiments with different HFM coatings. Laminins (10 µg/mL, LM332 and LM511) from Biolamina (Sundbyberg Stockholm, Sweden) ± Collagen Type IV (25 µg/mL) were diluted in PBS containing magnesium and calcium, according to manufacturer’s recommendations. HFMs were coated with the laminin containing solutions in low-binding Eppendorfs (Eppendorf, 0030108116), placed on a rocker and incubated at 37**°**C for 2h. Subsequently, HFMs were transferred to the Eppendorf containing cell suspension for seeding. For fixation, HFMs were washed 1x with HBSS and incubated in 4% formaldehyde (VWR, 9713.1000) for 45 minutes at 4℃. After incubation, HFMs were washed 1x with PBS (Sigma Aldrich, 32160405) and stored in PBS at 4℃ until immunofluorescence staining. HFMs were briefly transferred to a petri dish containing a roughly 1-2ml of HBSS to allow cutting to a suitable length for transfer to a 48 well plate without the HFM drying out. All following steps were carried out with volumes of 200μl per well. HFMs were washed 3x5 minutes on rocker in PBS-tween 0.1%. HFMs were then incubated for 10 minutes on rocker at RT in permeabilization buffer (0.3% triton). HFMs were incubated in blocking buffer (2% FCS, 2% w/v BSA in 0.1% PBS-tween) for 1 hour on rocker at RT. For HFMs stained only for DAPI (Invitrogen, D3571) and conjugated phalloidin 488 (Abcam, AB176753), each was diluted 1 in 1000 in blocking buffer and incubated with HFMs for 1 hour on rocker at RT in the dark. HFMs were washed 3x5 minutes in PBS-tween 0.1%. All samples were mounted on a wilco dish in Dako fluorescence Mounting Media (DAKO, S3023). Samples were stored at 4℃ in aluminium foil until imaged.

**References**

Bai J, Pu X, Zhang Y, Dai E (2022) Renal tubular gen e biomarkers identification based on immune infiltrates in focal segmental glomerulosclerosis. Ren Fail 44:966-986

Barnes DA, Firman JW, Belfield SJ, Cronin MTD, Vinken M, Janssen MJ, Masereeuw R (2024) Development of an adverse outcome pathway network for nephrotoxicity. Arch Toxicol 98:929-942

Bhargava P, Schnellmann RG (2017) Mitochondrial energetics in the kidney. Nat Rev Nephrol 13:629-646

Caetano-Pinto P, Stahl SH (2023) Renal Organic Anion Transporters 1 and 3 In Vitro: Gone but Not Forgotten. Int J Mol Sci 24:

Cao C, Ma Q, Huang X, Li A, Liu J, Ye J, Gui Y (2021) Targeted Demethylation of the PLOD2 mRNA Inhibits the Proliferation and Migration of Renal Cell Carcinoma. Front Mol Biosci 8:675683

Chuman L, Fine LG, Cohen AH, Saier MH, Jr. (1982) Continuous growth of proximal tubular kidney epithelial cells in hormone-supplemented serum-free medium. Journal of Cell Biology 94:506-510

Chung SD, Alavi N, Livingston D, Hiller S, Taub M (1982) Characterization of primary rabbit kidney cultures that express proximal tubule functions in a hormonally defined medium. Journal of Cell Biology 95:118-126

Courjault-Gautier F, Chevalier J, Abbou CC, Chopin DK, Toutain HJ (1995) Consecutive use of hormonally defined serum-free media to establish highly differentiated human renal proximal tubule cells in primary culture. Journal of the American Society of Nephrology 5:1949-1963

Detrisac CJ, Sens MA, Garvin AJ, Spicer SS, Sens DA (1984) Tissue culture of human kidney epithelial cells of proximal tubule origin. Kidney International 25:383-390

Faria J, Calcat ICS, Skovronova R, Broeksma BC, Berends AJ, Zaal EA, Bussolati B, O'Brien T, Mihăilă SM, Masereeuw R (2023) Mesenchymal stromal cells secretome restores bioenergetic and redox homeostasis in human proximal tubule cells after ischemic injury. Stem Cell Res Ther 14:353

Hoogstraten CA, Jacobs MME, de Boer G, van de Wal MAE, Koopman WJH, Smeitink JAM, Russel FGM, Schirris TJJ (2023) Metabolic impact of genetic and chemical ADP/ATP carrier inhibition in renal proximal tubule epithelial cells. Arch Toxicol 97:1927-1941

Kamiyama M, Garner MK, Farragut KM, Kobori H (2012) The establishment of a primary culture system of proximal tubule segments using specific markers from normal mouse kidneys. International Journal of Molecular Sciences 13:5098-5111

Kanehisa M (2019) Toward understanding the origin and evolution of cellular organisms. Protein Sci 28:1947-1951

Kanehisa M, Furumichi M, Sato Y, Matsuura Y, Ishiguro-Watanabe M (2025) KEGG: biological systems database as a model of the real world. Nucleic Acids Res 53:D672-d677

Kanehisa M, Goto S (2000) KEGG: kyoto encyclopedia of genes and genomes. Nucleic Acids Res 28:27-30

Lan R, Geng H, Singha PK, Saikumar P, Bottinger EP, Weinberg JM, Venkatachalam MA (2016) Mitochondrial Pathology and Glycolytic Shift during Proximal Tubule Atrophy after Ischemic AKI. J Am Soc Nephrol 27:3356-3367

Lin NYC, Homan KA, Robinson SS, Kolesky DB, Duarte N, Moisan A, Lewis JA (2019) Renal reabsorption in 3D vascularized proximal tubule models. Proceedings of the National Academy of Sciences of the United States of America 116:5399-5404

Nyhan KC, Faherty N, Murray G, Cooey LB, Godson C, Crean JK, Brazil DP (2010) Jagged/Notch signalling is required for a subset of TGFβ1 responses in human kidney epithelial cells. Biochim Biophys Acta 1803:1386-1395

Pettersen IKN, Tusubira D, Ashrafi H, Dyrstad SE, Hansen L, Liu XZ, Nilsson LIH, Løvsletten NG, Berge K, Wergedahl H, Bjørndal B, Fluge Ø, Bruland O, Rustan AC, Halberg N, Røsland GV, Berge RK, Tronstad KJ (2019) Upregulated PDK4 expression is a sensitive marker of increased fatty acid oxidation. Mitochondrion 49:97-110

Schley G, Klanke B, Schödel J, Kröning S, Türkoglu G, Beyer A, Hagos Y, Amann K, Burckhardt BC, Burzlaff N, Eckardt KU, Willam C (2012) Selective stabilization of HIF-1α in renal tubular cells by 2-oxoglutarate analogues. Am J Pathol 181:1595-1606

Sens DA, Detrisac CJ, Sens MA, Rossi MR, Wenger SL, Todd JH (1999) Tissue culture of human renal epithelial cells using a defined serum-free growth formulation. Experimental Nephrology 7:344-352

Shao X, Xu H, Kim H, Ljaz S, Beier F, Jankowski V, Lellig M, Vankann L, Werner JN, Chen L, Ziegler S, Kuppe C, Zenke M, Schneider RK, Hayat S, Saritas T, Kramann R (2024) Generation of a conditional cellular senescence model using proximal tubule cells and fibroblasts from human kidneys. Cell Death Discov 10:364

Tan L, Tang Y, Li H, Li P, Ye Y, Cen J, Gui C, Luo J, Cao J, Wei J (2021) N6-Methyladenosine Modification of LncRNA DUXAP9 Promotes Renal Cancer Cells Proliferation and Motility by Activating the PI3K/AKT Signaling Pathway. Front Oncol 11:641833

Taub M, Chuman L, Saier MH, Jr., Sato G (1979) Growth of Madin-Darby canine kidney epithelial cell (MDCK) line in hormone-supplemented, serum-free medium. Proceedings of the National Academy of Sciences of the United States of America 76:3338-3342

Taub N, Livingston D (1981) The development of serum-free hormone-supplemented media for primary kidney cultures and their use in examining renal functions. Annals of the New York Academy of Sciences 372:406-421

Wang V, Davis DA, Haque M, Huang LE, Yarchoan R (2005) Differential gene up-regulation by hypoxia-inducible factor-1alpha and hypoxia-inducible factor-2alpha in HEK293T cells. Cancer Res 65:3299-3306

Yang Y, Mihajlovic M, Valentijn F, Nguyen TQ, Goldschmeding R, Masereeuw R (2022) A Human Conditionally Immortalized Proximal Tubule Epithelial Cell Line as a Novel Model for Studying Senescence and Response to Senolytics. Front Pharmacol 13:791612
